# *HOTAIR* Promotes Prostate Cancer Progression by facilitating CDK9-Pol II Association and Transcriptional Elongation

**DOI:** 10.7150/ijbs.134766

**Published:** 2026-09-10

**Authors:** Yuyang Qian, Bo Wang, Guangsong Su, Peng Li, Zhenhao Zhao, Yuan Jiang, Lei Zhang, Zhongfang Zhao, Mulin Jun Li, Jiandang Shi, Dandan Huang, Wange Lu

**Affiliations:** 1 State Key Laboratory of Medicinal Chemical Biology, Frontiers Science Center for Cell Responses, College of Life Sciences, Nankai University, Tianjin, PR China.; 2 Department of Pharmacology, State Key Laboratory of Experimental Hematology, The Province and Ministry Co-sponsored Collaborative Innovation Center for Medical Epigenetics, Tianjin Key Laboratory of Inflammation Biology, School of Basic Medical Sciences, Tianjin Medical University, Tianjin, PR China.; 3 Department of Laboratory Medicine and Institute of Precise Medicine, The First Affiliated Hospital, Sun Yat-sen University, Guangzhou, PR China.; 4 Department of Head and Neck Oncology, Tianjin Medical University Cancer Institute &Hospital, Key Laboratory of Basic and Translational Medicine on Head & Neck Cancer, Tianjin, National Clinical Research Center for Cancer, Tianjin's Clinical Research Center for Cancer, Tianjin, PR China.

**Keywords:** *HOTAIR*, AR, CDK9, Pol II

## Abstract

Previous research has predominantly focused on the regulatory role of lncRNA *HOTAIR* in gene silencing by interacting with the Polycomb repressive complex 2 (PRC2), but its direct role as a transcriptional activator remains less well characterized. Here, our study reveals a novel regulatory mechanism of *HOTAIR*, which facilitates prostate cancer (PCa) progression by enhancing transcription elongation. Integrating ChIRP-seq, RNA-seq, and ChIP-seq, we found that HOTAIR co-localizes with AR and facilitates the interaction between CDK9 and Pol II. This function requires the 1-442 nt region and intact RNA, as deletion or RNase treatment disrupts the interaction. Re-expression of full-length HOTAIR, but not the deletion mutant, restores nascent transcription of target genes; conversely, CDK9 inhibition suppresses this elongation effect. We identified *MMP14* and *TNFAIP2* as functional downstream targets; ectopic expression of either rescues colony formation and invasive migration upon *HOTAIR* depletion. *HOTAIR* knockdown reduced Ser2P and Ser5P signals at target loci, yet RNA pull-down showed no direct binding to Pol II Ser5P, implying that any effect on initiation is indirect. This elongation activity operates in both AR-positive and AR-negative cells, with AR acting as a context-specific recruiter. Collectively, these findings support a model in which *HOTAIR* functions as an elongation activator and suggest potential therapeutic targets in prostate cancer.

## Introduction

Long non-coding RNAs (lncRNAs), a class of RNA transcripts exceeding 200 nucleotides, have drawn considerable attention due to their role in regulating gene expression, both locally (in *cis*) and across chromosomes (in *trans*) 1-4. The lncRNA HOX transcript antisense RNA (*HOTAIR*) is particularly noteworthy 5. Located at the *HOXC11* locus in the *12q13.13* region of the human genome, *HOTAIR* encodes a 2.2 kb transcript with 6 exons 6 and has been implicated in various cancers, including hepatocellular carcinoma (HCC), breast, colorectal, pancreatic, lung, and ovarian cancers 7-13. *HOTAIR* has a widespread impact on the progression of tumors, promoting tumor cell proliferation, invasion, metastasis, and enhancing drug resistance 14-16. Among the various malignancies, *HOTAIR*'s role is profound in PCa, one of the most lethal cancers in men 17. *HOTAIR* contributes to PCa progression by promoting androgen-independent AR activation and by binding to AR to prevent its ubiquitination-mediated degradation 18. Moreover, *HOTAIR* promotes PCa cell invasion by downregulating HEPACAM 19 and acts as a sponge for microRNAs, thereby activating STAT3 signaling and increasing PCa stem cell-like cells 20. *HOTAIR* is also implicated in high Gleason grade and neuro-metastatic PCa 21, 22.

A key regulatory function of *HOTAIR* is its interaction with the PRC2 and the histone H3K4 demethylase LSD1/CoREST/REST complex, acting as a gene repressor. By associating with these complexes, *HOTAIR* modulates chromatin compartments, inhibiting gene expression via regulation of histone H3 lysine 27 and lysine 4 methylation 23. Yet, recent studies have unveiled *HOTAIR*'s ability to activate gene expression. For instance, *HOTAIR* has been shown to promote adipose depot-specific target gene expression through preferential binding at transcription ends 24. Additionally, *HOTAIR* reportedly regulates NF-κB activation by inhibiting Iκ-Bα, leading to prolonged NF-κB activation and downstream gene expression following DNA damage 13. *HOTAIR*'s reach extends beyond transcriptional regulation to the post-translational level, where it interacts with ubiquitin ligases like E3 Dzip3 and its substrate ataxin-1, influencing ubiquitin-mediated proteolysis 25. Despite these advances, significant research gaps persist. The extent of *HOTAIR*'s transcriptional regulation, chromatin remodeling roles, and its genome-wide location, especially in the context of PCa, remain incompletely understood. The canonical pathway involving *HOTAIR*'s interaction with PRC2 in cancer progression is recognized, yet a more comprehensive understanding of *HOTAIR*'s multifaceted roles could provide critical insights into PCa diagnosis and treatment, thus illuminating potential therapeutic strategies.

To this end, we comprehensively examined the complex role of *HOTAIR* in PCa cell transcriptional regulation. Our research has revealed that *HOTAIR* exhibits a dual function in transcriptional regulation, depending on its binding to genomic regions enriched with different chromatin modifications. It can activate transcription in regions with low H3K27me3 enrichment in conjunction with AR, a function that is opposite to its role in regions with high H3K27me3 signals. In addition, we have uncovered a new mechanism for transcriptional elongation regulation, demonstrating that *HOTAIR* can facilitate the interaction between Pol II and CDK9, thereby promoting transcriptional elongation. This research provides new insights into the intricate crosstalk between *HOTAIR* and the epigenetic landscape in PCa cells, uncovering a unique capacity of *HOTAIR* to regulate gene expression depending on its genomic distribution.

## Materials and Methods

### Cell culture

The prostate cancer cells (22Rv1, C4-2B, PC-3) were cultured in RPMI 1640 (Gibco) supplemented with 10% FBS (BI) and 1% Penicillin/Streptomycin (Invitrogen). The cells were grown at 37℃ in an incubator with 5% CO_2_.

### 5' rapid amplification of cDNA ends (5' RACE)

To reveal the exact transcript of the *HOTAIR*, 5' RACE System for Rapid Amplification of cDNA Ends (Invitrogen, 18374058) was used. Prior to the reaction, total RNAs were extracted from cultured cells using TRIzol (Life Technologies) in 22Rv1, C4-2B and PC-3. cDNA synthesis was conducted according to the manufacturer's protocol with 500 ng of RNA. Gene specific primers were designed for PCR and nested PCR reactions. Final products were outsourced for Sanger sequencing (Genewiz, China). Primers used for 5' RACE are listed in Additional file 2: Table S2.

### CRISPR/Cas9-mediated *HOTAIR* poly-A insertion in PCa cells

A sgRNA targeting the second exon of *HOTAIR* was designed using the CRISPOR (https://crispor.gi.ucsc.edu/). The sgRNA plasmid, which carries a puromycin resistance cassette, and a donor oligonucleotide containing the poly-A signal sequence were co-transfected into 22Rv1 cells. To enrich for successfully transfected cells, cultures were treated with puromycin for 48 hours, followed by culture in regular medium for an additional two weeks to allow single colony formation.

For positive clone validation, individual colonies were picked and expanded. Genomic DNA was extracted from each clone and PCR was performed using primers flanking the insertion site. PCR products were resolved by agarose gel electrophoresis and compared with those from wild-type clones; clones showing a ~49 bp size shift were considered candidate positive for poly-A insertion. Candidate clones were further confirmed by Sanger sequencing using the same flanking primers. RT-qPCR was additionally performed on verified clones. All primer and sgRNA sequences are provided in Additional file 2: Table S2.

### Soft agar assay

The culture plates were filled with a medium containing 0.6% agarose (Lonza), which was then solidified at 4°C. Subsequently, 5000 cells were plated onto the solid agarose along with a medium containing 0.3% agarose. Every 3-4 days, the medium was changed, and the cells were cultured for 2-3 weeks. After staining the cell colonies with 0.005% crystal violet, the number of colonies was determined.

### Transwell assay

Cell invasion assays were conducted using Transwell plates (8 μm, Corning Costar). The upper chambers of the plates were precoated with Matrigel (BD), while the lower chambers were filled with 600 μl of complete 1640 medium containing 10% FBS. Subsequently, the upper chambers were seeded with 200 μl of serum-free medium containing (1 × 10^5^) cells. After two days, the cells were fixed with methanol, and then 0.1% crystal violet was added for 10 minutes. The migrated cells were then counted under a microscope.

### Chromatin isolation by RNA purification (ChIRP)

ChIRP was carried out in accordance with a published protocol with minor modifications 26. Briefly, 22Rv1 cells (2 × 10^7^) were crosslinked with 1% glutaraldehyde at room temperature for 10 minutes and then quenched with 0.125 M glycine for another 5 minutes. The cell pellets were weighed and suspended in 1 ml of full lysis solution (10 mM EDTA, 50 mM Tris-HCl pH 7.0, 1% SDS, 1× protease inhibitors, 500 U RNase inhibitor) for every 100 mg of cell pellets, and then sheared with a Bioruptor (Diagenode) at 4°C to obtain 100-500 bp fragments. Sheared chromatin was verified by agarose gel electrophoresis to confirm fragment sizes. The sonicated cell lysates were centrifuged and split into two 1 ml aliquots, mixed with 2 ml of the complete hybridization buffer (750 mM NaCl, 1 mM EDTA, 1% SDS, 50 mM Tris-HCl pH 7.0, 15% formamide, 1× protease inhibitors, 1000 U RNase inhibitor). The odd and even probe pools were then used for separate hybridization, which was carried out at 37 °C for 4 hours. After hybridization, beads were washed five times with wash buffer (2× SSC, 0.5% SDS) at 37°C. Following washing and elution, the eluted DNA was purified by 45 minutes of proteinase K digestion at 50°C. The NEBNext^®^ Ultra^™^ II DNA Library Prep Kit (NEB) was utilized in the construction of the DNA sequencing library. The purified DNA was subjected to next-generation sequencing and ChIRP-qPCR analysis. The biotin-labeled probes and qPCR primer sequences are listed in Additional file 2: Table S2.

### ChIRP-seq data analysis

Clean reads from the odd-probe, even-probe and input libraries, including biological replicates, were aligned to the human reference genome (GRCh38/hg38) using STAR 27. Reads with a mapping quality score below 30 and duplicated reads were removed using the view and rmdup functions of SAMtools 28, respectively. Normalized bigWig tracks for the odd-probe, even-probe and merged ChIRP-seq datasets were generated using the published ChIRP-seq processing pipeline 26.

Candidate HOTAIR-binding peaks were identified from the merged ChIRP-seq data using MACS2 29, and peaks overlapping ENCODE blacklist regions were excluded. Odd/even probe reproducibility was assessed by comparing the signal profiles of the odd- and even-probe libraries within each candidate peak and calculating the Pearson correlation between their binned signal intensities. Candidate peaks were retained as confident HOTAIR-binding regions when they showed strong odd/even reproducibility (Pearson's *r* > 0.8), significant enrichment (FDR < 0.05) and more than twofold read coverage relative to the input control. Genomic regions without detectable HOTAIR ChIRP-seq coverage were defined as HOTAIR-negative regions. Confident HOTAIR peaks and their nearby genes were annotated using HOMER 30, and the resulting gene annotations were used for downstream visualization, including volcano and box plots.

### Matched HOTAIR-negative background selection

HOTAIR-positive bins were retained from the original HOTAIR ChIRP peak-associated regions. HOTAIR-negative background bins were sampled from non-peak genomic regions after excluding HOTAIR peaks, blacklist/problematic intervals, and regions failing basic sequence filters. Candidate regions were matched to HOTAIR-positive bins by chromosome, 2-kb region length, GC-content bin, and log_10_ distance-to-nearest-TSS bin. Matching quality was evaluated by GC-content distributions, TSS-distance distributions, chromosome counts, and summary tests.

### Chromatin immunoprecipitation (ChIP)

22Rv1 cells (1 × 10^7^ per assay) were cross-linked with 1% formaldehyde for 10 minutes at room temperature and quenched with 0.125 M glycine for 5 minutes. The cell pellets were lysed and sonicated to yield 200-500 bp DNA fragments. Sheared DNA fragments were confirmed by agarose gel electrophoresis. Each sonicated lysate was then centrifuged and immunoprecipitated with antibodies against Pol II Ser2P (Abcam, ab193468), Pol II Ser5P (Abcam, ab5131), BRD4 (CST, 13440S), H3K27ac (CST, 8173S), H3K27me3 (CST, 9733S), and H3K4me1 (Abcam, ab8895) at 4°C overnight with rotation. 2-5 µg of each antibody was used per immunoprecipitation. Subsequently, 30 μl of protein G beads were added and incubated for an additional 2 hours. The beads were washed five times and eluted in TE buffer containing 1% SDS. The eluted DNA was de-crosslinked using Proteinase K at 65°C for 2 hours. DNA purification was performed using DNA Clean & Concentrator kits (Zymo Research). The purified DNA was subjected to next-generation sequencing and ChIP-qPCR analysis. The qPCR primer sequences are listed in Additional file 2: Table S2.

### Cleavage under targets and tagmentation (CUT & Tag)

CUT & Tag was performed using the Hyperactive Universal CUT & Tag Assay Kit (Vazyme, TD903). In brief, 22Rv1 cells (1 × 10^5^) were used for the CUT & Tag experiment of AR. Before proceeding, cells were examined under a microscope to confirm viability (>90%) and the absence of clumping. Cells were incubated with concanavalin A-coated beads at room temperature for 10 minutes, followed by overnight incubation with the primary AR antibody (CST, 5153S) at 4°C. The secondary antibody was then incubated at room temperature for 1 hour. Finally, pA/G-Tn5 transposase was applied to cut the genomic DNA and add a specific adaptor sequence to create the DNA sequencing library. Libraries were purified and analyzed on an Agilent Bioanalyzer to confirm fragment size distribution and quantify library concentration prior to sequencing. All experiments were performed with two biological replicates.

### ChIP-seq and CUT & Tag data analysis

ChIP-seq datasets were uniformly processed using the AQUAS pipeline 31, whereas CUT&Tag datasets were processed according to the published CUT&Tag workflow 32. Briefly, clean reads were aligned to the human reference genome (GRCh38/hg38), and only uniquely mapped reads were retained for downstream analyses. Quality control was performed at both the individual-library and replicate levels. Sequencing depth, mapping rate, fraction of reads in peaks (FRiP) or corresponding enrichment metrics, peak reproducibility, and genome-wide signal correlations were evaluated for the H3K27ac, H3K27me3, Pol II Ser2P, Pol II Ser5P, and AR datasets. Replicate concordance was assessed using Pearson correlation coefficients calculated from genome-wide binned signal profiles, together with the other dataset-specific quality-control metrics. Replicates showing consistently high concordance and satisfactory overall data quality were merged for downstream peak calling and visualization.

Peaks associated with histone modifications were called using MACS2 29, whereas transcription-factor peaks were identified using SPP 33. Peaks overlapping ENCODE blacklist regions were excluded. Genomic annotation of the retained peaks was performed using HOMER 30 with default parameters.

Normalized coverage tracks in bigWig format were generated from the processed alignments using bamCoverage in deepTools 34 with the parameters --binSize 100, --normalizeUsing RPGC, --effectiveGenomeSize 2862010578, and --extendReads. The resulting normalized signal profiles were summarized using computeMatrix and visualized as heatmaps and metaplots using plotHeatmap and plotProfile, respectively.

### Heatmap comparing ChIRP-seq, ChIP-seq and CUT & Tag signals

To visualize and compare HOTAIR, AR, Pol II Ser2P, Pol II Ser5P, BRD4, H3K4me1, H3K27me3 and H3K27ac signals across the genome, we first segmented the genome into bins with a bin size of 2 kb. Each normalized coverage track (bigWig) file was used to calculate the signal in each bin using the binnedAverage function of GenomicRanges 35, an R package. For better visualization, we log_2_-transformed all signals and used pheatmap, an R package, to cluster bins and plot the heatmap.

### RNA pull-down assay

Biotin-labeled RNAs were transcribed *in vitro* using a T7 Quick High Yield RNA Synthesis Kit (NEB) and Biotin-16-UTP (Roche), treated with RNase-free DNase I (Promega), and purified using RNA Clean & Concentrator-25 (Zymo Research). For each binding assay, 2 μg of RNA was mixed with a nuclear extract from 22Rv1, C4-2B and PC-3 cells (1 × 10^7^) and incubated at 4°C for 2 hours while rotating. A biotin-labeled antisense RNA fragment was used as a negative control. Subsequently, 50 μl of washed streptavidin beads (Invitrogen) were added to each assay and incubated for 45 minutes at 4°C. After three washes with washing buffer (25 mM Tris-HCl, pH 7.5, 5 mM EDTA, 0.1% NP-40, 150 mM KCl, 0.5 mM DTT, 1 × Protease inhibitor, and 100 U/ml RNase inhibitor), the pull-down samples were boiled for 10 minutes at 100°C in 1 × protein loading buffer. The samples were then subjected to detection by a western blot assay. The primers used for cloning templates to transcribe truncated *HOTAIR* RNA variants of different lengths *in vitro* are listed in Additional file 2: Table S2.

### RIP assay

22Rv1 cells (2 × 10^7^) were harvested and re-suspended in 1 ml of RIPA buffer (150 mM NaCl, 1 mM EDTA, 50 mM Tris-HCl, pH 7.5, 0.1% SDS, 1% NP-40, 0.5% sodium deoxycholate, 1 × PMSF, and 0.5 mM DTT, supplemented with 100 U/mL RNase inhibitor). After being incubated on ice for 30 minutes with regular vortexing, the supernatants were collected by centrifugation at 12,000 RPM for 10 minutes at 4°C. Subsequently, 4 μg of Pol II Ser2P antibody (Abcam, ab193468), Pol II Ser5P (Abcam, ab5131), AR antibody (CST, 5153S), CDK9 (Santa Cruz, sc-13130) and the corresponding IgG were separately added to the supernatant and incubated at 4°C for 4 hours. Next, 40 μl of Protein G beads (Invitrogen) were added, and the mixture was incubated for 2 hours at 4°C. After washing four times with RIPA buffer, the RNA-protein complex was digested with Proteinase K at 50°C for 45 minutes, and then RNA was extracted with 200 μl TRIzol. For RT-qPCR, reverse transcription was performed with a PrimeScript^™^ RT reagent kit with gDNA eraser (Takara, RRO47A) followed by standard RT-qPCR analysis. 10% of the lysate was saved as input for normalization. The RIP-qPCR primers are listed in Additional file 2: Table S2.

### Co-IP assay

Cell pellets were lysed in IP lysis buffer (150 mM NaCl, 50 mM Tris-HCl pH 7.5, 1 mM EDTA, 1% NP-40, 1 × PMSF, and 1 × protease inhibitor (Roche)). The cell lysates were pre-cleared with protein G beads at 4°C for 1 hour with rotation. An aliquot of the pre-cleared lysates was used as input, and the remainder was incubated with CDK9 and Pol II antibodies (Santa Cruz, sc-13130 and sc-47701) or the corresponding IgG at 4°C overnight on a rocker. Next, protein G beads were added and incubated at 4°C for another 2 hours. The beads were washed 4 times with IP lysis buffer and boiled with 1 × protein loading buffer. Western blot analysis was performed using Pol II and CDK9 antibodies.

### Nascent RNA capture assay

A Click-iT™ Nascent RNA Capture Kit (Invitrogen, C10365) was used to detect nascent RNA synthesis according to the manufacturer's instructions. PCa cells were subjected to the indicated treatments and subsequently incubated with 0.4 mM 5-ethynyl uridine (EU, an alkyne-modified uridine analog) for 6 h at 37°C. Following EU labeling, total RNA labeled with EU was isolated, and the incorporated EU was conjugated to azide-modified biotin through a copper-catalyzed click reaction. The biotinylated nascent RNA transcripts were then captured using Dynabeads™ MyOne™ Streptavidin T1 magnetic beads (Invitrogen, 65601) and extensively washed to remove unlabeled RNA. The captured nascent transcripts were used as a template for reverse transcriptase-mediated cDNA synthesis, and the resulting cDNA was analyzed by qPCR to determine the relative abundance of nascent transcripts. The nascent RNA qPCR primers are listed in Supplementary Table S2.

### CDK9 kinase-dependent functional assay

To determine whether the rescue of transcriptional elongation was dependent on CDK9 catalytic activity, wild-type PCa cells and *HOTAIR*-silenced cells rescued with full-length HOTAIR, the Δ1-442 mutant, or an empty vector were treated with 50 nM AZD4573 (MCE, HY-112088), a selective CDK9 inhibitor, or an equivalent volume of DMSO as the vehicle control. Following treatment, nascent RNA was labeled and isolated as described above, and the abundance of nascent target transcripts was quantified by qPCR. Nascent transcript levels were normalized to *18S rRNA* and expressed relative to the DMSO-treated sample within each condition. The effect of CDK9 inhibition was calculated as the ratio of nascent transcript abundance in AZD4573-treated cells to that in the matched DMSO-treated cells (CDK9i/DMSO). The nascent RNA qPCR primers are listed in Supplementary Table S2.

### RNase sensitivity assay

To determine whether the interaction between CDK9 and Pol II was dependent on RNA, an RNase sensitivity assay was performed in combination with Co-IP. PCa cells subjected to the indicated treatments were lysed in immunoprecipitation buffer supplemented with protease and phosphatase inhibitors, and cell lysates were divided into two equal aliquots. One aliquot was treated with 50 μg/mL RNase A (Thermo Scientific, EN0531) under the indicated conditions, whereas the other was incubated with an equivalent volume of RNase-free buffer as the untreated control. Following RNase A treatment, Co-IP was subsequently performed as described above. The immunoprecipitated complexes were analyzed by western blot.

### CRISPRi assay

The CRISPRi sgRNAs targeting the* HOTAIR* promoter were cloned into the lenti-hU6-sgRNA-dCas9-KRAB-T2a-Puro plasmid. The CRISPRi lentiviral particles were generated in 293T cells using the core plasmid (mentioned above), along with the psPAX2 and pMD2.G packaging plasmids (Addgene, 12260 and 12259). Viral supernatants were collected at 48 hours post-transfection, filtered through 0.45 µm filters, and used immediately or stored at -80°C. C4-2B and PC-3 cells were transduced with the lentiviral supernatants. After 24 hours, the medium was replaced with fresh medium containing puromycin, and selection was maintained for 2 days. Following selection, cells were expanded in puromycin-free medium for 3-5 days, then harvested for validation and downstream experiments. Knockdown efficiency was assessed by RT-qPCR. The sgRNAs were designed using the online sgRNA design tool (https://portals.broadinstitute.org/gpp/public/analysis-tools/sgrna-design). All sgRNAs are listed in Additional file 2: Table S2.

### CRISPRa assay

Lentiviral particles were generated in 293T cells using the lentiMPHv2 plasmid (Addgene, 89308) along with the packaging plasmids psPAX2 and pMD2.G (Addgene, 12260 and 12259). Viral supernatants were collected at 48 hours post-transfection, filtered through 0.45 µm filters, and used to transduce the established *HOTAIR* CRISPRi C4-2B and PC-3 cell lines. Transduced cells were selected with hygromycin for 7 days. The CRISPRa sgRNAs targeting the promoter regions of *MMP14* and *TNFAIP2* were designed and cloned into the lentiSAMv2 plasmid (Addgene, 75112). Lentiviral particles were generated using the sgRNA-containing lentiSAMv2 plasmid along with the same packaging plasmids. Viral supernatants were collected and filtered as above, and used to transduce the hygromycin-resistant cell lines. After 24 hours, the cells were selected with blasticidin for 5 days. Following selection, cells were expanded and harvested for downstream experiments. Activation efficiency was assessed by RT-qPCR. The sgRNAs were designed using the online sgRNA design tool (https://portals.broadinstitute.org/gpp/public/analysis-tools/sgrna-design). All CRISPRa sgRNA sequences are listed in Additional file 2: Table S2.

### RNA isolation and RT-qPCR

Total RNAs were extracted from cultured cells using TRIzol (Life Technologies) following the manufacturer's instructions. Subsequently, cDNAs were reverse transcribed from the total RNA using a PrimeScript^™^ RT reagent kit with a gDNA Eraser (Takara, RRO47A). RT-qPCR was performed using FastStart Universal SYBR Green mix (Roche) and a Bio-Rad iQ5 System. Relative gene expression was quantified using comparative CT and normalized to *HPRT1* mRNA. The RT-qPCR primers are shown in Additional file 2: Table S2.

### RNA-seq data analysis

RNA-seq datasets were analyzed using the TOPMed RNA-seq pipeline 36. Differentially expressed genes were identified using DESeq2 based on default parameters 37. To generate more accurate fold-change estimates, the lfcShrink function of DESeq2 was used to correct fold-change values of genes showing low expression. Genes with an absolute log_2_ fold change >1 and an adjusted *P*-value <0.05 were considered significantly differentially expressed. ComplexHeatmap 38 was used to visualize differentially expressed genes. Gene ontology biological process enrichments were performed using clusterProfiler 39.

### Western blot

Cell pellets were lysed in RIPA buffer supplemented with a protease inhibitor cocktail (Roche). The protein concentration was measured using a bicinchoninic acid assay. Subsequently, the proteins were boiled, separated on an SDS-polyacrylamide gel, and transferred to a PVDF membrane (Millipore). The membranes were blocked in BSA and incubated with primary antibodies at 4°C overnight. Then, a secondary antibody was added and incubated at room temperature for 1 hour. The western blot bands were detected using an ECL Western Blotting Detection System (GE). The same membrane was stripped and re-incubated with different primary antibodies for the detection of different proteins. Stripping was performed using Fast Western Blot Antibody Stripping Buffer (Vazyme, E701) for 10-15 minutes at room temperature with gentle agitation. The stripped membrane was extensively washed three times in TBST and incubated with the next primary antibody.

### Re-expression of direct targets of *HOTAIR*

The coding sequences (CDS) of the two *HOTAIR* targets (*MMP14* and *TNFAIP2*) were individually cloned into the pLVX-3 × flags-IRES-puromycin vectors. The empty pLVX-3 × flag-IRES-puromycin vector was used as a control. Subsequently, the plasmids containing the CDS of the target genes and the empty vector control were transfected into the *HOTAIR*-silenced 22Rv1 cells. Puromycin was then used to select the cells that stably expressed the *HOTAIR* targets. The stable overexpression of the targets was validated by western blot analysis.

### Tumor xenograft assay in nude mice

For analysis, seven-week-old male BALB/c-Nude mice were purchased from Gempharmatech (Nanjing, China, stock for mouse strain: #D000521). The animals were maintained under specific pathogen-free conditions on a 12-hour light-to-dark cycle and ad libitum access to food and water. Prior to mice injection, 5 × 10^6^ 22Rv1 cells were cultured and harvested for subcutaneous injection with 50 μl matrigel (Corning, 354262) in the right flank region. Mice were randomly divided into 4 groups as follows: 22Rv1 WT group, *HOTAIR* Poly-A knock-in group, HKI+MMP14 overexpression group and HKI+TNFAIP2 overexpression group. Each group had 5 mice. Tumor growth was evaluated with an electronic caliper and measured every 3 days until day 21 and subsequently removed. Tumor volume was calculated using the formula: (length × width^2^)/2. Tumor weight was measured on an analytical balance. Animal experiments were performed following protocols approved by the Clinical Research and Laboratory Animal Ethics Committee, The First Affiliated Hospital of Sun Yat-sen University.

### Statistical analysis

All data are represented as means ± SEM. Two-tailed Student's *t* test was used for statistical analysis (*P < 0.05, **P < 0.01, ***P < 0.001; ns, not significant).

## Results

### *HOTAIR* promotes the progression of PCa through significant dual regulation of gene expression

Firstly, to identify the predominant transcript isoform of *HOTAIR* in prostate cancer, we performed 5' rapid amplification of cDNA ends (RACE) experiments using the human prostate cancer cell line 22Rv1, C4-2B and PC-3. Our RACE analysis revealed that the NR_186241.1 transcript variant represents the major expressed isoform of *HOTAIR* in these prostate cancer cell models (Additional file 1: Fig. S1A-B). Next, to assess the functional role of *HOTAIR* in PCa, we utilized CRISPR/Cas9 technology to knock in a 49 bp poly-A transcription termination signal at the second exon of* HOTAIR* in 22Rv1 cells, effectively blocking *HOTAIR* transcription* in situ* (Fig. 1A). The insertion was validated through genomic PCR and RT-qPCR (referred to as HKI-1 and HKI-2) (Fig. 1B-C). We then investigated the impact of this *HOTAIR* poly-A knock-in on the tumorigenic and invasive capabilities of the 22Rv1 cells. Using a soft agar assay, we observed a marked reduction in tumor formation capability in both HKI-1 and HKI-2 lines as compared to the wild-type (WT) controls (Fig. 1D). This was further supported with a Transwell assay, which revealed a decrease in cell invasion in the *HOTAIR* poly-A knock-in lines (Fig. 1E). To further clarify the essential role of* HOTAIR* in the malignant progression of prostate cancer, we also conducted tumor-related phenotype experiments in two other PCa cell lines (C4-2B, PC-3) using the CRISPRi-mediated *HOTAIR* silencing. The results showed that there was a significant inhibition of colony formation and a reduction in invasive migration in *HOTAIR*-silenced C4-2B and PC-3 cells compared with the WT cells (Additional file 1: Fig. S2A-F). To further confirm that the observed phenotypic defects were specifically caused by *HOTAIR* silencing, we performed rescue experiments in the *HOTAIR* poly-A knock-in cells. Notably, ectopic expression of *HOTAIR* significantly restored the impaired clonogenic capacity and invasive migration potential of these tumor cells (Additional file 1: Fig. S3A-C). Collectively, these results suggest that *HOTAIR* plays a critical role in PCa progression.

We then performed RNA-seq on WT 22Rv1 cells and the *HOTAIR* poly-A knock-in lines (HKI-1 and HKI-2) to examine the transcriptional alterations induced by *HOTAIR*'s silencing. We observed that the number of significantly down-regulated genes was approximately 2.5-fold greater than that of the up-regulated genes (Fig. 1F). To identify direct *HOTAIR* targets on a genome-wide scale, we conducted ChIRP-seq in 22Rv1 WT cells, identifying 13,446 genomic loci potentially bound by HOTAIR (Additional file 1: Fig. S4A-B). Our analysis of these loci showed HOTAIR's propensity to bind to exons, introns, and promoters, with a notable bias towards gene bodies over promoters (Fig. 1G-H). By integrating RNA-seq and ChIRP-seq data, we identified 132 significantly up-regulated and 261 significantly down-regulated genes as potential direct *HOTAIR* targets (Fig. 1I). Interestingly, a considerable proportion of its direct targets was actually down-regulated, demonstrating a previously unappreciated activating role of *HOTAIR*. Gene Ontology (GO) enrichment of these down-regulated direct targets indicated a significant association with cell-substrate adhesion (Fig. 1J), concordant with the phenotypic changes observed in the *HOTAIR* poly-A knock-in cells. This evidence points towards a bivalent role for *HOTAIR* in gene regulation, with capabilities to both activate and repress its target genes, thus suggesting noncanonical functions of *HOTAIR* in modulating gene expression and PCa progression.

### *HOTAIR* is significantly linked to the enrichment of H3K27ac and H3K27me3 in the PCa genome

Previous studies have established *HOTAIR*'s role in transcriptional silencing, but emerging evidence suggests its potential involvement in gene activation 18, 24, 40. To explore how the genome-wide chromatin landscape changes upon *HOTAIR* silencing in PCa cells, we performed ChIP-seq of two active histone marks (H3K27ac and H3K4me1) and one repressive mark (H3K27me3) in two *HOTAIR* poly-A knock-in lines (HKI-1, HKI-2) and 22Rv1 WT controls (Additional file 1: Fig. S5A-B). While H3K4me1 enrichment did not show significant change, both H3K27me3 and H3K27ac levels were altered following *HOTAIR* silencing (Additional file 1: Fig. S6A-C). Given the relevance of H3K27me3 to repressive chromatin, we focused on changes in H3K27me3 enrichment around HOTAIR target regions. We categorized HOTAIR-associated regions into H3K27me3-positive (H3K27me3+) and H3K27me3-negative (H3K27me3-) groups based on baseline H3K27me3 enrichment in WT cells (Fig. 2A). Most H3K27me3+ regions overlapped with HOTAIR target regions, consistent with the reported repressive function of *HOTAIR* 26. However, within this group, not all regions showed increased H3K27ac upon *HOTAIR* silencing, some displayed reduced H3K27ac instead, suggesting repressive tendencies (Fig. 2A). The H3K27me3- group also included regions with HOTAIR target regions, where reduced H3K27ac was observed upon *HOTAIR* silencing. Furthermore, a portion of the H3K27me3- group was found within the HOTAIR negative target region. These regions are likely indirectly regulated by *HOTAIR* and showed repression after *HOTAIR* silencing (Fig. 2A).

In light of changes in gene expression in either the H3K27me3+ or H3K27me3- group around HOTAIR target regions upon *HOTAIR* silencing, we examined the correspondence between H3K27ac changes and transcriptional outcomes (Fig. 2B). In the H3K27me3+ group around HOTAIR target regions, the proportions of up- and downregulated genes were comparable upon *HOTAIR* silencing, which is consistent with the bidirectional H3K27ac changes in this group. In the H3K27me3- group around HOTAIR target regions, the majority of genes were downregulated, consistent with the reduced H3K27ac observed in Figure 2A. These observations indicate that *HOTAIR* regulates gene expression in a context-dependent manner. Furthermore, genes in the H3K27me3- group around the HOTAIR negative target region also showed a significant decrease in expression after *HOTAIR* silencing, suggesting that *HOTAIR* might indirectly activate gene expression in some cases (Additional file 1: Fig. S7A-B). As for the genomic distribution of HOTAIR target regions, our analysis showed that the H3K27me3+ group around HOTAIR target regions was mainly distributed on promoters, while the H3K27me3- group around HOTAIR target regions was mostly found on gene bodies (Fig. 2C). This suggests *HOTAIR* might play dual roles depending on the level of different signal enrichment on promoter regions, while primarily functioning as an activator on gene bodies (Fig. 2D). Together, these data suggest that *HOTAIR* exerts context-dependent regulatory effects depending on the baseline H3K27me3 context, with gene expression changes generally consistent with the corresponding H3K27ac alterations.

### *HOTAIR* mediates the co-binding of AR/Pol II to activate transcription

Given our preliminary findings indicating a novel role of *HOTAIR* in promoting transcription, we sought to further examine this function in PCa cells. We integrated ChIRP-seq of HOTAIR and collected ChIP-seq of PCa associated important transcription factors for colocalization analysis (Additional file 2: Table S1 and Fig. S8A-B). We found that HOTAIR co-locates with AR, Pol II and BRD4 (Additional file 2: Table S1). To dissect the role of co-localized transcription factors with HOTAIR in transcriptional regulation, independent CUT&Tag of AR combined with ChIP-seq of Pol II Ser2P (the form of Pol II during transcription elongation), Pol II Ser5P (the form of Pol II during transcription initiation), and BRD4 (a reader of histone acetylation) were performed in both wild-type and *HOTAIR* poly-A knock-in lines (Additional file 1: Fig. S5A-B). The AR signal decreased around the center of the HOTAIR peak when *HOTAIR* was silenced (Additional file 1: Fig. S9). Consequently, our attention was primarily drawn to the changes in AR peaks. Our results indicated that, although the number of AR peaks remained relatively stable after silencing *HOTAIR*, their genomic distribution exhibited significant changes: AR peaks around HOTAIR target regions such as promoters, exons, and introns decreased, while those located in HOTAIR-negative target regions, such as intergenic regions, increased (Fig. 3A). Moreover, despite an overall decrease in the AR signal in all genomic regions after *HOTAIR* was silenced, the AR signal in HOTAIR target regions showed a more pronounced decrease (Fig. 3B, Additional file 1: Fig. S10A-B). This suggests that *HOTAIR* is involved in modulating AR-dependent transcriptional regulation.

We also observed that although the overall Pol II Ser2P and Pol II Ser5P signals did not change significantly after silencing *HOTAIR* (Additional file1: Fig. S9), their signals decreased around *HOTAIR* targets and in regions where AR signaling decreased after *HOTAIR* was silenced (Fig. 3C). By dividing AR signaling into two groups (AR-down and others) based on changes in AR enrichment upon *HOTAIR* silencing, we found that the AR-down group showed a significant decrease in Pol II Ser2P and Pol II Ser5P enrichment, but the enrichment of BRD4 did not exhibit remarkable alterations. In addition, the “others” group only exhibited a significant increase in Pol II Ser5P enrichment after* HOTAIR* silencing (Fig. 3C-D). These results suggest that *HOTAIR* may be involved in transcription regulation together with AR/Pol II. To further confirm that AR signals changed in conjunction with Pol II Ser2P and Pol II Ser5P after *HOTAIR* silencing, we integrated ChIP-seq data of AR/Pol II Ser2P/Pol II Ser5P for a combined analysis. We observed that Pol II Ser2P and Pol II Ser5P enrichment decreased along with AR signals after *HOTAIR* was silenced, particularly around HOTAIR target regions (Fig. 3E). Together, these results suggest that *HOTAIR* may promote transcription in association with AR/Pol II.

### *HOTAIR* activates transcription by associating with elongation factor CDK9

To further elucidate the potential role of *HOTAIR* in transcription regulation in PCa cells, we conducted RNA pull-down experiments using three PCa cell lines to investigate the interactions between HOTAIR, AR, and Pol II. Compared with HOTAIR antisense control, the 5'-terminal fragment (1-442 nt) of HOTAIR sense RNA specifically pulled down Pol II Ser2P but not Pol II Ser5P. This suggests that *HOTAIR* may play a direct role in transcription elongation (Fig. 4A-C). The RNA pull-down experiment also showed the 5'-terminal fragment (1-442 nt) of HOTAIR specifically interacted with both AR and CDK9 (Fig. 4A-C). CDK9, being the primary component of the positive transcription elongation factor b (P-TEFb), is known to phosphorylate Pol II at the serine 2 site, thereby promoting transcriptional elongation. Subsequently, we performed RNA Immunoprecipitation (RIP) in three PCa cell lines to further investigate potential specific interactions between HOTAIR and Pol II, AR and CDK9. Our results showed significant enrichment of HOTAIR by immunoprecipitation of Pol II Ser2P, AR and CDK9 (Fig. 4D), further confirming these interactions. We also performed Co-immunoprecipitation (Co-IP) of CDK9 and Pol II in three PCa cell lines, and found that CDK9 associated with Pol II, and the association was significantly reduced in *HOTAIR*-silenced cells (Fig. 4E-G).

To determine whether HOTAIR directly bridges CDK9 and Pol II, we performed RNase-treated Co-IP experiments. RNase treatment markedly reduced the CDK9-Pol II interaction compared to the untreated control, indicating that an intact RNA molecule, presumably HOTAIR, is required for stabilizing the complex (Fig. 4H-J). We also conducted rescue experiments in three *HOTAIR*-silenced PCa cell lines by reintroducing full-length HOTAIR or a mutant lacking nucleotides 1-442 (Δ1-442). Full-length HOTAIR restored the CDK9-Pol II interaction, whereas the Δ1-442 mutant failed to do so (Fig. 4K-M and Additional file1: Fig. S11A-C). To assess the functional relevance of this scaffolding activity, we examined the phenotypic consequences of HOTAIR rescue in *HOTAIR*-silenced cells. Full-length HOTAIR, but not the Δ1-442 mutant, rescued colony formation and invasive migration (Additional file1: Fig. S11D-I). In AR-positive 22Rv1 and C4-2B cells, AR knockdown significantly reduced the CDK9-Pol II interaction, indicating that AR is required for *HOTAIR*'s scaffolding function in this context (Fig. 4N-O and Additional file1: Fig. S12A-B). Together, these results support a model in which HOTAIR facilitates the CDK9-Pol II association to promote transcriptional elongation.

### Integrative epigenomic analysis reveals a novel function of *HOTAIR* in chromatin activation

To further understand how *HOTAIR* regulates gene activation, we analyzed alterations in H3K27ac and AR enrichment post-*HOTAIR* silencing in H3K27me3+ and H3K27me3- categories. In H3K27me3+ regions, *HOTAIR* silencing led to increased H3K27ac at promoters, consistent with a repressive role of *HOTAIR* in this context (Fig. 5A). Proceeding with this line of inquiry, we further divided the H3K27me3- category into AR-down and other subsets based on the observed changes in AR enrichment following *HOTAIR* silencing. In the AR-down subset, HOTAIR target regions showed reduced H3K27ac upon *HOTAIR* silencing, suggesting that HOTAIR may facilitate chromatin activation through AR at these loci, particularly around all HOTAIR target regions and on gene bodies marked by low H3K27me3 enrichment. By contrast, the other subsets remained mostly unchanged, aside from some activation within 1 kb of the promoter region (Fig. 5A). To evaluate *HOTAIR*'s potential role in gene activation within H3K27me3- regions, we integrated all markers (AR, Pol II Ser2P, Pol II Ser5P, H3K27me3, and H3K27ac) for analysis, and divided the H3K27me3- region into H3K27ac-down and H3K27ac-up subsets, depending on the changes in H3K27ac signal enrichment following *HOTAIR* silencing. The H3K27ac-down subset displayed reduced enrichment of AR, Pol II Ser2P, and Pol II Ser5P around H3K27me3- regions; however, the H3K27ac-up subset did not show a significant decrease (Fig. 5B). This suggests that HOTAIR may interact with AR and Pol II to activate genes, particularly on H3K27me3- regions. Moreover, the AR-down subset demonstrated a decrease in gene expression on promoters around H3K27me3- regions (Fig. 5C). Further analysis comparing changes in the enrichment of Pol II Ser2P, Pol II Ser5P, and AR revealed that downregulated genes around HOTAIR target regions had lower enrichment of AR, Pol II Ser2P and Pol II Ser5P compared to HOTAIR-negative target regions (Fig. 5D-E).

Additionally, our observations on two key cancer-related genes (*MMP14* and *TNFAIP2*) 41, 42 are consistent with the regulation of the H3K27me3- group around HOTAIR target regions (Fig. 6A-B). In the RNA-seq volcano plot, *MMP14* and *TNFAIP2* were labeled to show their positions. Both genes were enriched in cell-substrate adhesion-related GO terms, consistent with the observed phenotypic changes (Additional file 1: Fig. S13A-B). ChIP-qPCR and ChIRP-qPCR analyses demonstrated that *HOTAIR* silencing significantly reduced the enrichment of AR, H3K27ac, Pol II Ser2P, and HOTAIR at both gene loci (Fig. 6C-D). Furthermore, RT-qPCR verification showed both genes were downregulated after silencing *HOTAIR* in all three PCa cell lines (Fig. 6E).

To determine whether these two genes are direct targets of *HOTAIR*-mediated transcriptional elongation, we examined their nascent transcript levels in *HOTAIR*-silenced cells rescued with full-length HOTAIR or the Δ1-442 mutant. Full-length HOTAIR rescued nascent transcription of both genes, whereas the Δ1-442 mutant failed to do so (Fig. 6F). To test whether this effect depends on CDK9 catalytic activity, we treated the rescue groups with AZD4573, a selective CDK9 inhibitor. CDK9 inhibition caused a milder reduction in nascent transcripts in the full-length rescue group than in the Δ1-442 mutant or empty vector groups (Fig. 6G), suggesting that *HOTAIR* promotes elongation of these target genes through CDK9 kinase activity. In AR-positive 22Rv1 and C4-2B cells, AR knockdown significantly decreased nascent transcripts of both genes, indicating that AR is required for *HOTAIR*'s elongation function at these loci (Fig. 6H-I).

Moreover, we separately and stably reintroduced both genes into the *HOTAIR*-silenced lines of three PCa cell lines to determine whether they are responsible for the tumorigenesis defects observed in the *HOTAIR* silenced lines (Additional file 1: Fig. S14A-B). As confirmed by soft agar and Transwell assays, re-expression of *MMP14* and *TNFAIP2* significantly rescued the reduction in colony formation and cell invasion in the* HOTAIR* silenced 22Rv1 cells (Fig. 6J-M, Additional file 1: Fig. S14C-F). Similar results were observed in C4-2B and PC-3 cells (Additional file 1: Fig. S14G-N). To further evaluate the biological function of *HOTAIR* and its targets *in vivo*, we established subcutaneous xenograft models of 22Rv1 WT cells, *HOTAIR*-silenced cells and *HOTAIR*-silenced cells with ectopic expression of either *MMP14* or *TNFAIP2*. Consistent with our *in vitro* findings, *HOTAIR* silencing significantly inhibited tumor growth compared to WT controls. Importantly, overexpression of either MMP14 or TNFAIP2 in *HOTAIR*-silenced 22Rv1 cells substantially rescued tumor growth, reaching levels comparable to WT controls (Fig. 6N). Tumor growth curves and endpoint tumor weights demonstrated similar rescue effects (Fig. 6O-P). These data indicate that both genes are the direct functional targets of* HOTAIR* in the H3K27me3- context.

Finally, to explore the potential relevance of our findings to clinical prostate cancer, we performed a cross-dataset comparison by analyzing public AR, H3K27ac, and H3K27me3 ChIP-seq data from prostate tumors (GSE130408) together with our HOTAIR ChIRP-seq data. This analysis showed that HOTAIR-associated regions identified in our system overlap with tumor-associated AR-bound and H3K27ac-marked active chromatin regions, while a subset also overlaps with H3K27me3-marked repressive domains. Although these public tumor ChIP-seq datasets are not derived from matched patient samples and therefore do not provide direct clinical validation of *HOTAIR* function in tumors, the cross-dataset comparison is consistent with the possibility that HOTAIR-associated regulatory regions are connected to tumor-relevant chromatin features. Together with our mechanistic analyses, these observations suggest a potential role for *HOTAIR* in shaping AR/Pol II-associated transcriptional programs and repressive chromatin contexts in prostate cancer, which will require further validation in matched clinical samples or patient-level datasets (Additional file1: Fig. S15).

Taken together, our findings suggest that *HOTAIR* associates with AR/Pol II to promote gene expression in a context-dependent manner, thereby facilitating tumorigenesis (Fig. 7).

## Discussion

With increasing technological innovations, global analyses of RNA/chromatin interactions have been used to define roles of RNAs in gene regulation at various genomic locations 43. However, to our knowledge, few systematic studies have analyzed lncRNA/chromatin interactions as well as the associated epigenetic transcriptome 24, 44, 45. Here, we applied ChIRP-seq to redefine the genome-wide location of HOTAIR in PCa and found a different genomic distribution of HOTAIR than reported previously in breast cancer 26. Notably, our integrative multi-omics approach (combining ChIRP-seq, RNA-seq, and ChIP-seq) unveils *HOTAIR*'s dual roles in gene regulation, contingent on its specific genomic location. This integrative approach reshapes our understanding of *HOTAIR*'s functional versatility and underscores the complexity of lncRNA action. Our work highlights the potential of comprehensive epigenomic analysis in lncRNA research and lays the groundwork for future investigations into the multifaceted roles of lncRNAs in cancer progression.

Although it is well-established that *HOTAIR* represses gene expression through interactions with the PRC2 and the LSD1/CoREST/REST complex 23, its role as an activator remains incompletely understood 18, 40, 46. It has been found that *HOTAIR* stimulates transcription via the NF-κB pathway to induce immunoescape 46, drives transcription of the c-MYC target genes by acting as a scaffold for the HBXIP and LSD1 complex 40, and promotes transcription of AR-associated genes independent of androgen, contributing to the progression of CRPC 18. Despite these findings, a critical gap persists in our understanding of *HOTAIR*'s direct impact on gene transcription based on its genomic location. Our study addresses this gap, providing evidence that HOTAIR, interacting with AR/CDK9/Pol II, promotes transcriptional elongation. Notably, this elongation-promoting function is not limited to AR-positive contexts. A key question arising from our data is why *HOTAIR* retains this elongation-promoting activity in AR-null PC-3 cells despite the absence of AR. We propose that AR functions primarily as a recruiter rather than an integral component of the elongation machinery. In AR-positive cells, AR directs HOTAIR to its target chromatin sites, whereas in AR-negative cells, alternative transcription factors or lineage-specific regulators likely fulfill this recruitment role. Two observations support this interpretation. First, our RNA pull-down and RIP assays show that AR, CDK9, and Pol II all associate with the same 1-442 nt region of HOTAIR, suggesting that the effector machinery and its recruiter associate with a shared region. Second, HOTAIR promotes nascent transcription in PC-3 cells even in the absence of AR, implying that the core bridging function between CDK9 and Pol II does not require a specific upstream factor to bring HOTAIR to chromatin. This view is consistent with the established modularity of *HOTAIR* 7, 23, in which the same region that engages one set of effectors can, in a different cellular context, interact with alternative partners without compromising its core activity. Whether this AR-independent mechanism operates in AR-negative prostate tumors remains to be investigated. The potential of *HOTAIR* as a unique therapeutic target in cancer treatment, given its special positioning and function in gene regulation, merits further exploration.

Through the integration of ChIRP-seq and RNA-seq data, our research has identified significant cancer-promoting genes directly regulated by *HOTAIR*, highlighting the important role of *HOTAIR* in PCa. We unveiled key *HOTAIR* targets such as *MMP14* and *TNFAIP2*, both of which have critical implications in cancer progression 47-51. MMP14, a member of the matrix metalloproteinase family of proteases, is instrumental in angiogenesis and tumor invasion 47-49. *TNFAIP2*, regulated by NF-κB, promotes cell motility in nasopharyngeal carcinoma 50 and fosters proliferation and invasive migration in breast cancer cells 51. The identification of these *HOTAIR* targets underscores the potential of utilizing these genes in the diagnosis and treatment of PCa. Our findings pave the way for a more comprehensive understanding of *HOTAIR*'s role in cancer progression, emphasizing the importance of its epigenomic context in gene regulation.

## Conclusions

Our findings indicate that HOTAIR facilitates CDK9-Pol II interaction to promote transcriptional elongation. This function is AR-dependent in AR-positive cells but remains active in AR-null cells through alternative recruiters. Our data do not support a direct role in initiation. Taken together, these results support a context-dependent elongation mechanism for *HOTAIR* in prostate cancer.

## Supplementary Material

Supplementary figures and tables.

## Figures and Tables

**Figure 1:**
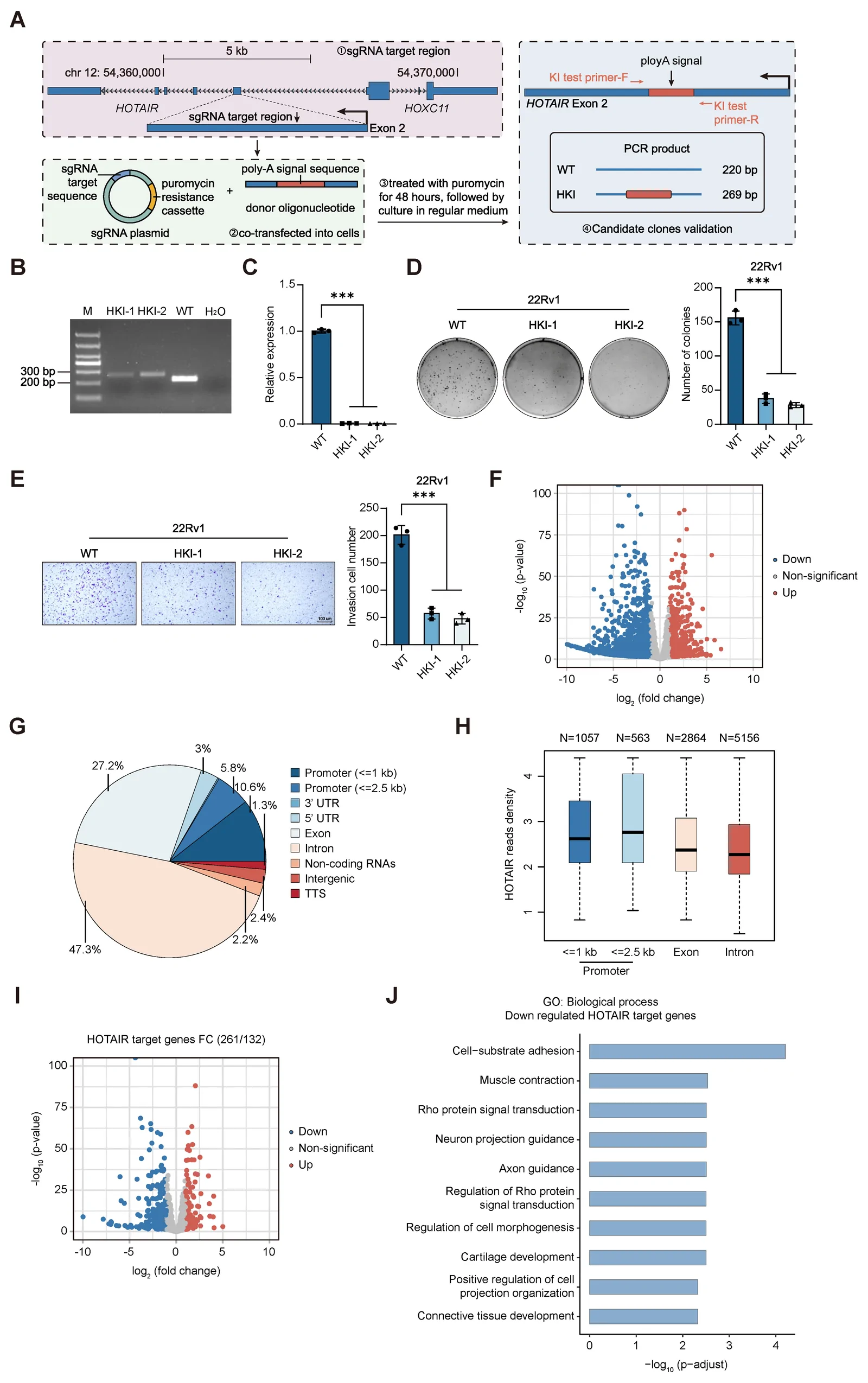
***HOTAIR* promotes PCa progression by regulating gene expression in a bivalent manner.** (**A**) Diagram showing CRISPR/Cas9-mediated knock-in of a 49 bp of poly-A transcription termination signal (red box) at the second exon of *HOTAIR*. Primers used for genotyping are indicated as orange arrows. (**B**) Genotyping PCR was performed using the genomic DNA isolated from the 22Rv1 WT and two poly-A knock-in lines (HKI-1, HKI-2), with H_2_O used as a negative control. “M” represents the DNA marker. (**C**) RT-qPCR comparing the expression levels of *HOTAIR* in the 22Rv1 WT cells and *HOTAIR* poly-A knock-in lines (HKI-1, HKI-2). Data are shown as means ± SEM of three independent experiments (***P<0.001). (**D**) Soft agar assay comparing colony formation in the 22Rv1 WT and *HOTAIR* poly-A knock-in cell lines (HKI-1, HKI-2). Quantification of colony number is shown as means ± SEM of three independent experiments (***P<0.001). (**E**) Transwell assays indicating invasive migration by the 22Rv1 WT and *HOTAIR* poly-A knock-in cell lines (HKI-1, HKI-2). Quantification of migrated cells is shown as means ± SEM of three independent experiments (***P<0.001). (**F**) Volcano plot showing significantly changed genes in the 22Rv1 WT versus *HOTAIR* poly-A knock-in cell lines (HKI-1 and HKI-2) (fold change>1 or <-1, padj<0.05). (**G**) Pie chart showing genomic distribution of 13,446 HOTAIR ChIRP peaks in the 22Rv1 WT cells. Peaks were annotated by HOMER. (**H**) Box plot showing genome-wide read density of HOTAIR ChIRP-seq peaks in the 22Rv1 WT cells. (**I**) Volcano plot showing significantly changed genes among *HOTAIR* direct targets after *HOTAIR* was silenced. (fold change > 1 or < -1, padj < 0.05). (**J**) GO analysis of significantly downregulated genes among *HOTAIR* direct targets after *HOTAIR* was silenced. The top 10 items are shown according to adjusted *p*-value. Statistical significance was assessed using two-tailed Student's *t* test. For sequencing data, statistical methods are described in Methods. See also Additional file 1: Figs. S1-S4.

**Figure 2:**
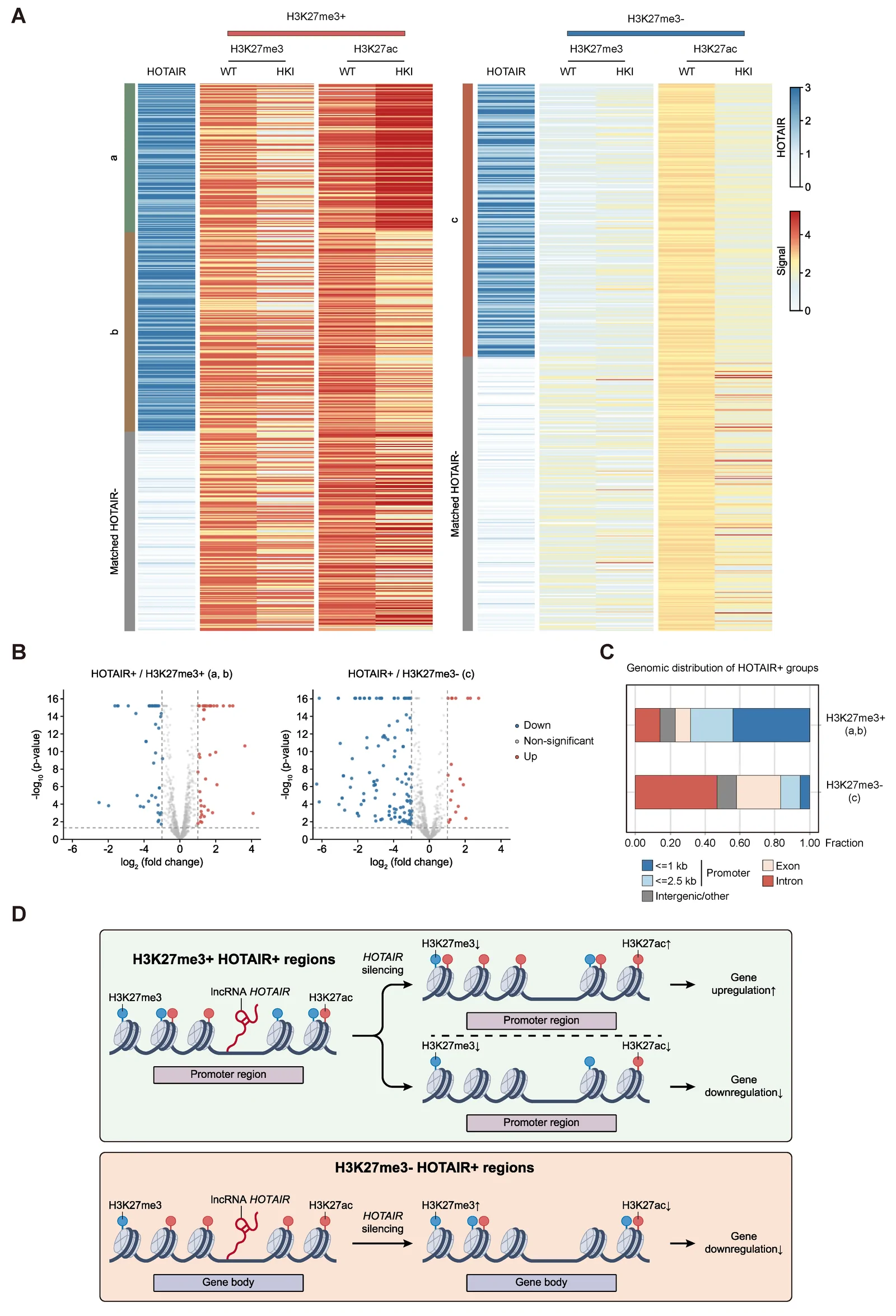
***HOTAIR* exerts context-dependent chromatin regulatory effects.** (**A**) Regions were first stratified by WT H3K27me3 signal into H3K27me3+ and H3K27me3- backgrounds. HOTAIR-positive regions were then divided into groups a, b, and c according to their H3K27ac response after *HOTAIR* knockdown. In each H3K27me3 background, matched HOTAIR-negative regions were selected as controls. The heatmaps show H3K27me3 and H3K27ac signals in WT and HKI samples, together with HOTAIR signal. (**B**) Volcano plots showing expression changes of genes targeted by these regions after *HOTAIR* silencing. (**C**) Genomic annotation of the HOTAIR-positive groups, showing the fraction of regions mapped to promoter-proximal, exonic, intronic, and intergenic/other categories. **(D)** Schematic summary of HOTAIR-mediated chromatin regulation. At promoters in H3K27me3-positive contexts, *HOTAIR* silencing results in bidirectional H3K27ac changes, correlating with either up- or downregulation of nearby genes. On gene bodies in H3K27me3-negative contexts, *HOTAIR* silencing leads to reduced H3K27ac and downregulated gene expression. All HKI data are pooled from two biological replicates. For sequencing data, statistical methods are described in Methods. See also additional file 1: Figs. S5-S7.

**Figure 3:**
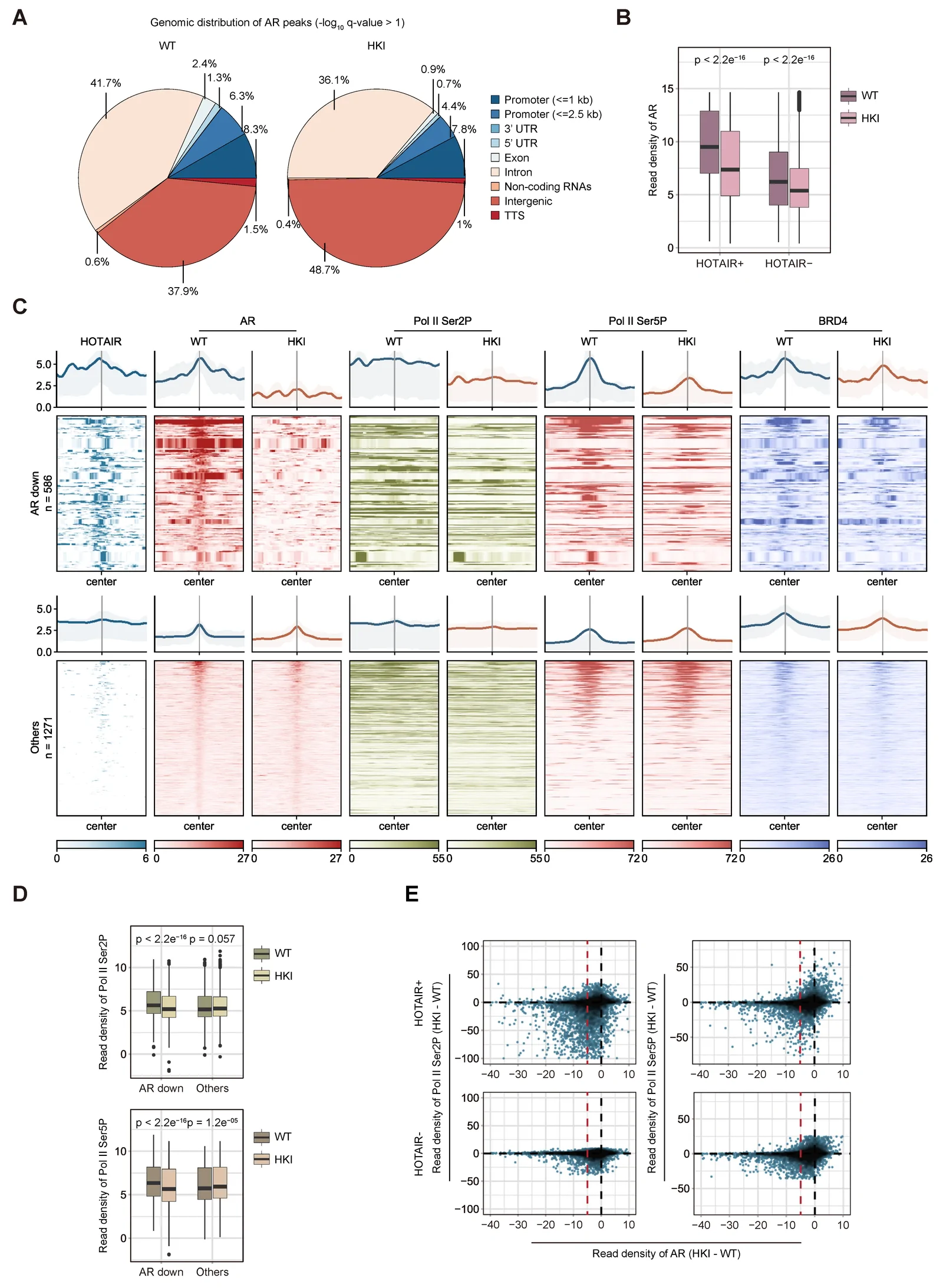
***HOTAIR* colocalizes with AR to promote transcription.** (**A**) Pie chart indicating genomic distribution of AR peaks in the 22Rv1 WT and HKI cell lines. Peaks were annotated by HOMER. (**B**) Boxplots comparing changes of AR signals between the 22Rv1 WT and HKI cell lines around the HOTAIR target and HOTAIR-negative target regions. (**C**) Heatmaps and average signal profiles showing HOTAIR ChIRP-seq and ChIP-seq signals for AR, Pol II Ser2P, Pol II Ser5P and BRD4 in 22Rv1 WT and HKI cells. Regions are centered on AR peaks and displayed within ±2.5 kb from the peak center. (**D**) Boxplots comparing changes of Pol II Ser2P and Pol II Ser5P signals between the 22Rv1 WT and HKI cell lines around “AR-down” and “others” groups after *HOTAIR* was silenced. Based on the changes in AR enrichment following *HOTAIR* silencing, we categorized AR signals into two groups: “AR-down” and “others”. (**E**) Scatter plot illustrating changes in the enrichment of Pol II Ser2P and Pol II Ser5P signals, along with the altered AR signals around the HOTAIR target and HOTAIR negative target regions following *HOTAIR* silencing. All HKI data represent pooled results from two biological replicates. For sequencing data, statistical methods are described in Methods. See also Additional file 1: Fig. S8-S10; Additional file 2: Table S1.

**Figure 4:**
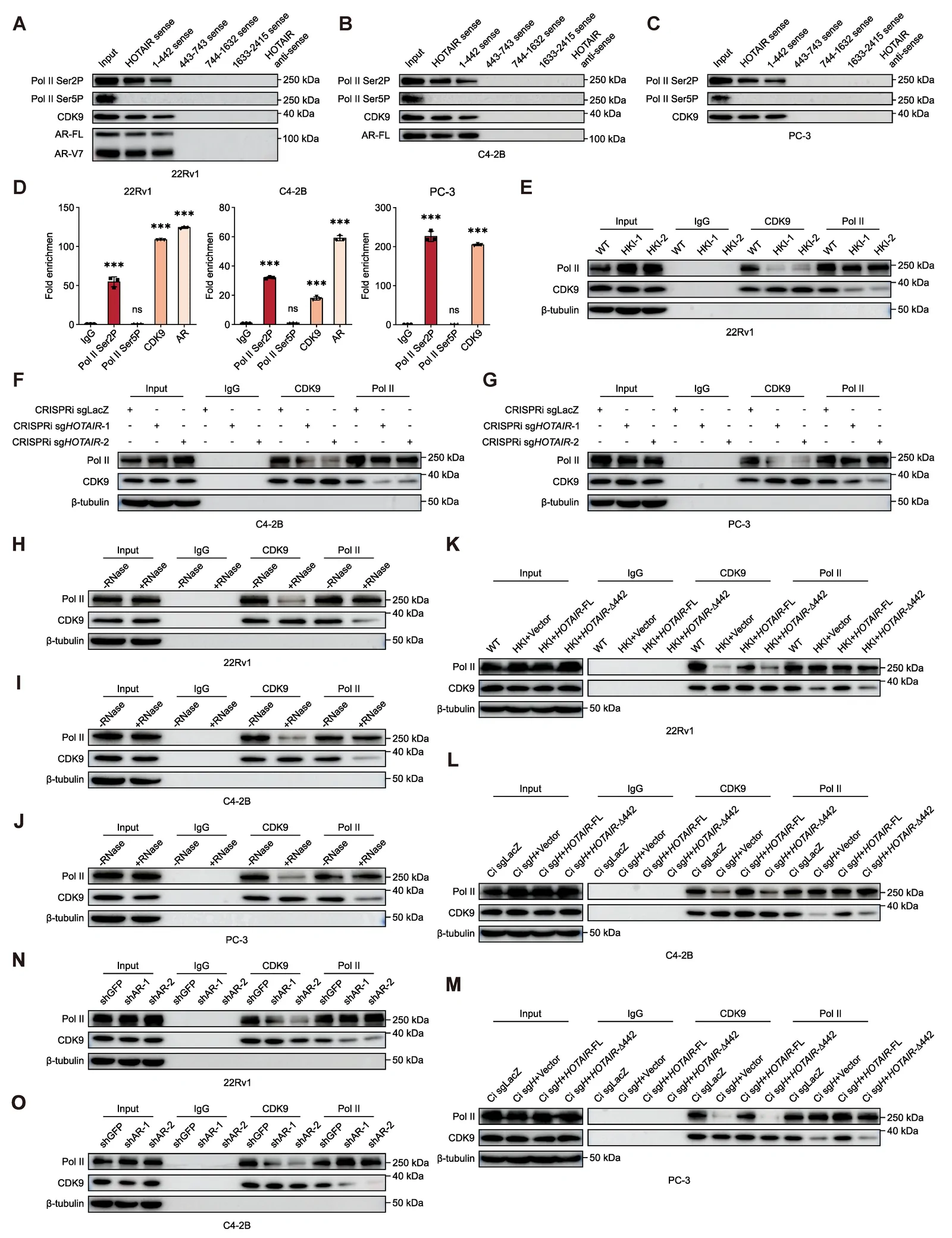
***HOTAIR* promotes transcription elongation by interacting with CDK9.** (**A-C**) Western blot analysis of proteins interacting with HOTAIR, including Pol II Ser2P, Pol II Ser5P, CDK9, and AR. RNA Pull-down assays were performed in the 22Rv1, C4-2B and PC-3 cells using biotin-labeled truncated *HOTAIR* RNA probe transcribed from the full-length sense *HOTAIR* gene, with an antisense *HOTAIR* probe as a negative control. (**D**) RT-qPCR analysis of HOTAIR enrichment by Pol II Ser2P, Pol II Ser5P, CDK9 and AR antibodies. RIP assays were carried out in the 22Rv1, C4-2B and PC-3 cells using antibodies against Pol II Ser2P, Pol II Ser5P, CDK9 and AR, with IgG as a control. Data are shown as means ± SEM of three independent experiments (***P<0.001). (**E-G**) Co-IP of CDK9 with Pol II in WT and *HOTAIR*-silenced PCa cell lines (22Rv1, C4-2B, and PC-3). Immunoprecipitation was performed using anti-CDK9 and anti-Pol II antibodies, followed by Western blotting with anti-Pol II and anti-CDK9 antibodies. (**H-J**) Co-IP of CDK9 with Pol II in 22Rv1, C4-2B and PC-3 cells following RNase treatment or untreated control. (**K-M**) Co-IP of CDK9 with Pol II in WT 22Rv1, C4-2B and PC-3 cells and *HOTAIR*-silenced cells rescued with empty vector, full-length HOTAIR, or Δ1-442 mutant. (**N-O**) Co-IP of CDK9 with Pol II in AR-positive 22Rv1 and C4-2B cells following AR knockdown or shGFP. Statistical significance was assessed using two-tailed Student's *t* test. See also Additional file 1: Fig. S11-S12.

**Figure 5:**
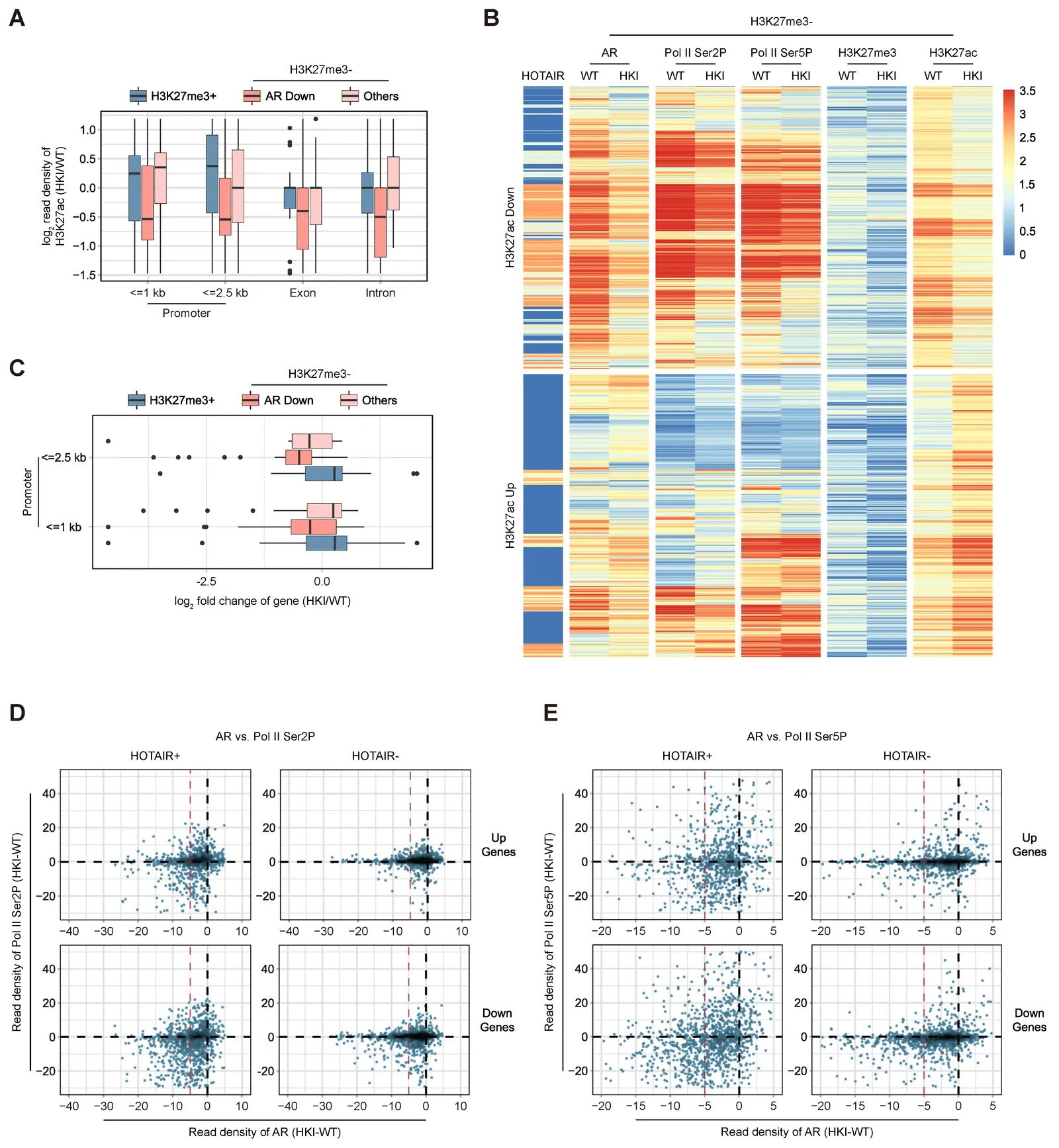
** The new role of *HOTAIR* in gene activation within H3K27me3- regions.** (**A**) Boxplots comparing changes in read density of H3K27ac peaks in “H3K27me3+”, “H3K27me3- and AR-down” and “H3K27me3- and others” groups around *HOTAIR* target regions after *HOTAIR* was silenced. We divided the H3K27me3- category into “AR-down” and “others” subsets based on the observed changes in AR enrichment following *HOTAIR* silencing. (**B**) Heatmaps showing changes in the enrichment of AR, Pol II Ser2P, Pol II Ser5P, H3K27me3, and H3K27ac after* HOTAIR* was silenced around the H3K27me3- region (signal is defined as the average read density of each 2-kb bin). Heatmaps were divided into H3K27ac-down and H3K27ac-up groups, depending on changes in the H3K27ac signal enrichment following *HOTAIR* silencing. (**C**) Boxplots comparing changes in gene expression on promoter regions based on the three groups defined in Fig. 5A. (**D**-**E**) Scatter plots depicting changes in the enrichment of Pol II Ser2P/Pol II Ser5P signals along with the changed AR signal around the HOTAIR target and negative target regions after *HOTAIR* was silenced. *HOTAIR* regulated genes were divided into up-regulated and down-regulated groups. All HKI data represent pooled results from two biological replicates. For sequencing data, statistical methods are described in Methods.

**Figure 6:**
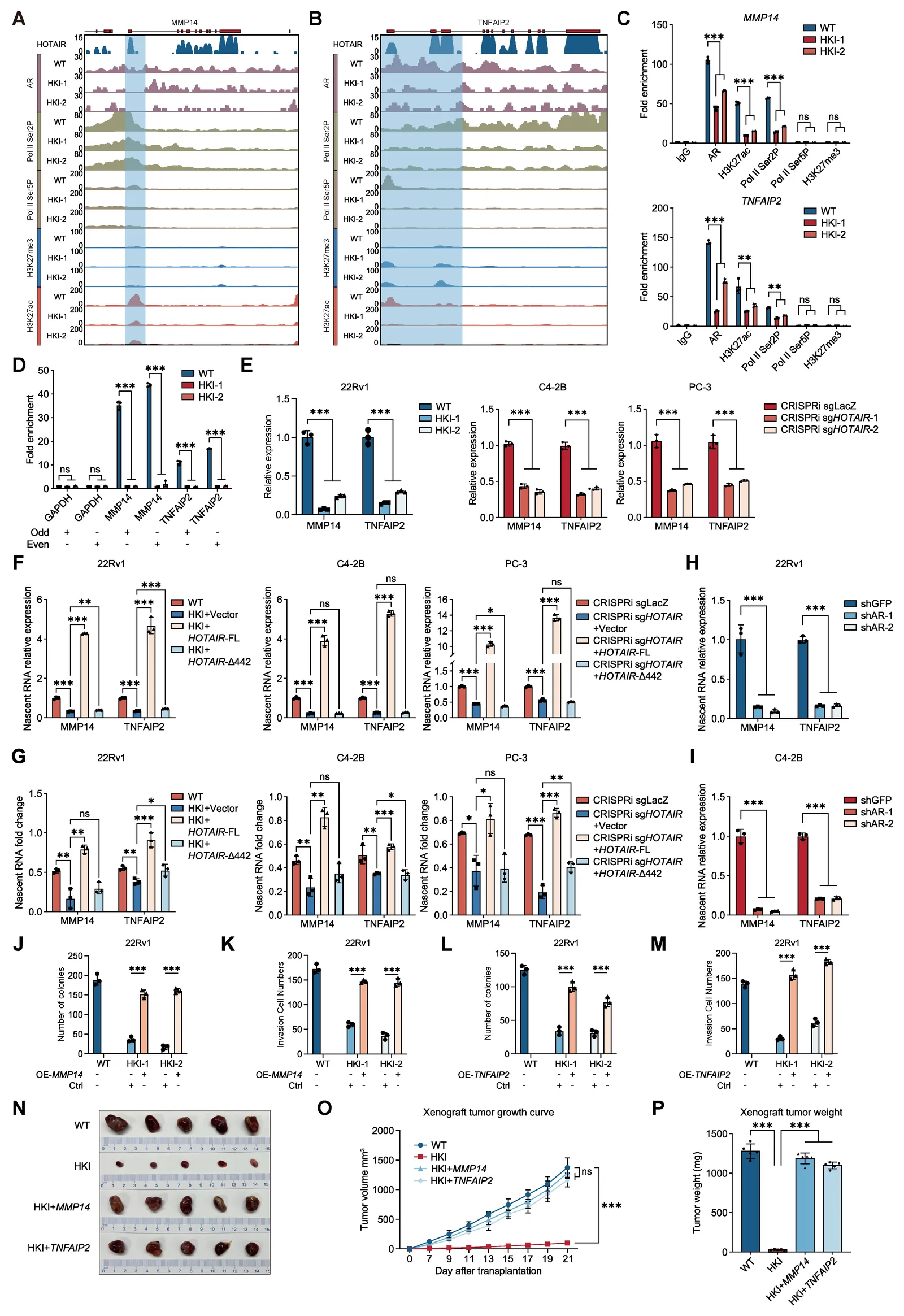
** MMP14 and TNFAIP2 promote PCa progression dependent on *HOTAIR*-mediated transcriptional elongation.** (**A**-**B**) Genome-browser screenshots showing changes in enrichment of AR, Pol II Ser2P, Pol II Ser5P, H3K27me3 and H3K27ac signals around two *HOTAIR* target genes (*MMP14* and *TNFAIP2*) in 22Rv1 WT versus *HOTAIR*-silenced cells (HKI-1, HKI-2). (**C**) ChIP-qPCR showing the enrichment of AR, H3K27ac, Pol II Ser2P, Pol II Ser5P and H3K27me3 marks at* MMP14* and *TNFAIP2* gene loci in 22Rv1 WT cells versus *HOTAIR*-silenced cells (HKI-1, HKI-2), with IgG as a negative control. Data are shown as means ± SEM of three independent experiments (***P<0.001). (**D**) ChIRP-qPCR showing the enrichment of HOTAIR at* MMP14* and *TNFAIP2* gene loci in 22Rv1 WT cells versus *HOTAIR*-silenced cells (HKI-1, HKI-2), with *GAPDH* as a negative control. “odd” and “even” indicate two independent sets of HOTAIR ChIRP probes. Data are shown as means ± SEM of three independent experiments (***P<0.001). (**E**) RT-qPCR showing the expression of* MMP14* and *TNFAIP2* in WT and *HOTAIR*-silenced lines of three PCa cell lines. Data are shown as means ± SEM of three independent experiments (***P<0.001). (**F**) Nascent transcript levels of *MMP14* and *TNFAIP2* in WT 22Rv1, C4-2B and PC-3 cells and *HOTAIR*-silenced cells reintroduced with an empty vector, full-length HOTAIR, or Δ1-442 mutant, measured by nascent RNA qPCR. Data are shown as means ± SEM of three independent experiments (*P<0.05, **P<0.01, ***P<0.001; ns, not significant). (**G**) Nascent transcript levels of *MMP14* and *TNFAIP2* in WT 22Rv1, C4-2B and PC-3 cells and *HOTAIR*-silenced cells reintroduced with an empty vector, full-length HOTAIR, or Δ1-442 mutant, followed by CDK9 inhibitor or DMSO treatment. Data are shown as means ± SEM of three independent experiments (*P<0.05, **P<0.01, ***P<0.001; ns, not significant). (**H-I**) Nascent transcript levels of *MMP14* and *TNFAIP2* in AR-positive 22Rv1 and C4-2B cells following AR knockdown or shGFP. Data are shown as means ± SEM of three independent experiments (***P<0.001). (**J, L**) Quantification data of the soft agar colony formation experiments for 22Rv1 WT cells, *HOTAIR*-silenced lines, and *HOTAIR*-silenced lines re-expressing MMP14 or TNFAIP2. Data are shown as means ± SEM of three independent experiments (***P<0.001). (**K, M**) Quantification data of the Transwell experiments for 22Rv1 WT cells, *HOTAIR*-silenced lines, and *HOTAIR*-silenced lines re-expressing MMP14 or TNFAIP2. Data are shown as means ± SEM of three independent experiments (***P<0.001). (**N**) Representative images of xenografts derived from 22Rv1 WT cells, *HOTAIR*-silenced cells, and *HOTAIR*-silenced cells with re-expressed MMP14 and TNFAIP2. (**O-P**) Growth curves and endpoint tumor weights of xenografts across groups (n = 5). Data are presented as means ± SEM (***P<0.001). Statistical significance was assessed using two-tailed Student's *t* test. For sequencing data, statistical methods are described in Methods. See also Additional file 1: Fig. S14-S15.

**Figure 7:**
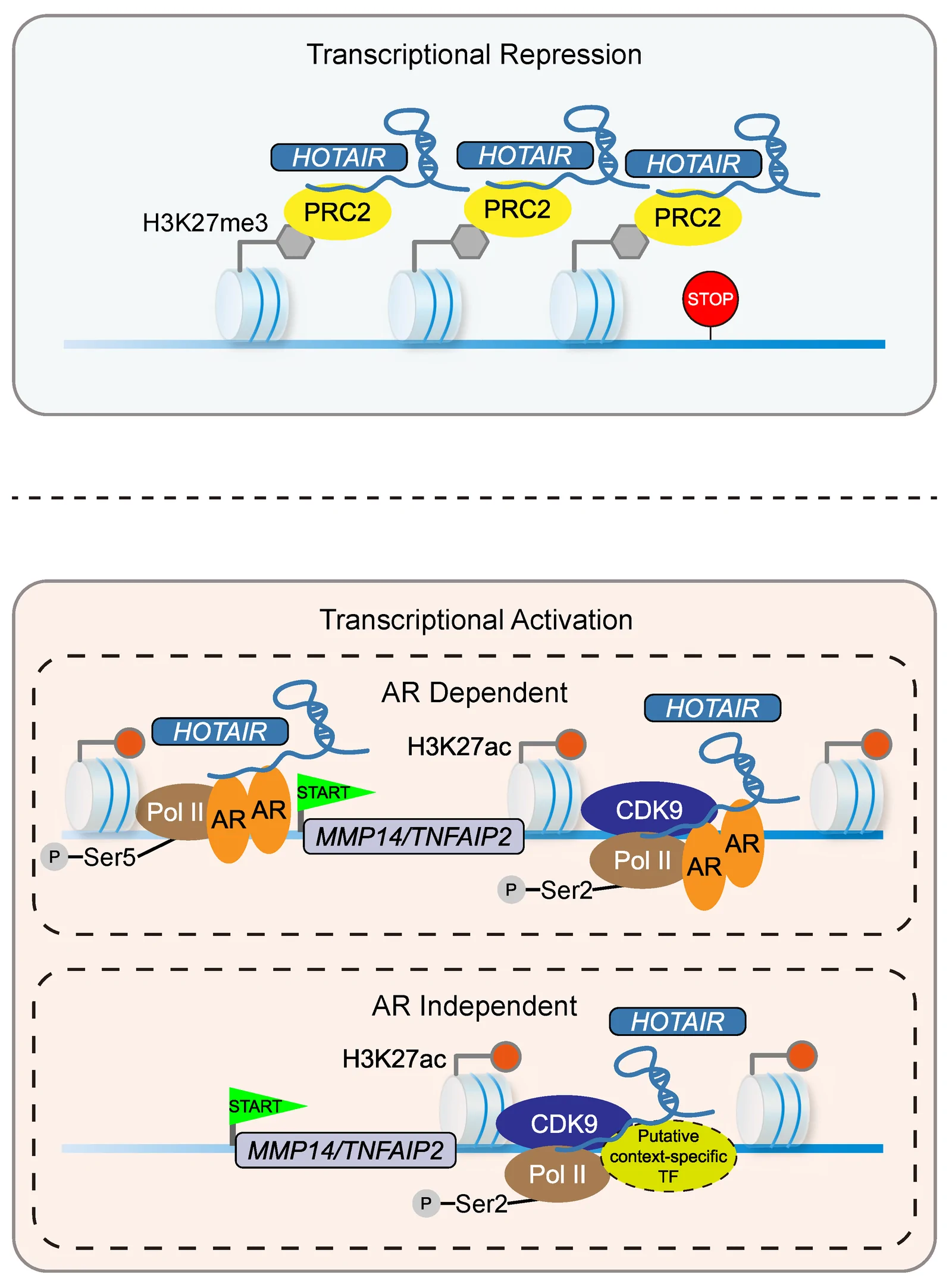
** Proposed model for *HOTAIR*-mediated transcriptional regulation in prostate cancer.** The upper panel shows the classical HOTAIR-PRC2 axis in transcriptional repression through H3K27me3 deposition. The lower panel describes the activation mechanism: in AR-positive cells, HOTAIR facilitates CDK9-Pol II interaction and sustains transcriptional elongation of target genes including *MMP14* and *TNFAIP2*. In AR-negative cells, other putative context-specific transcription factors may substitute for AR to serve this function.

## Data Availability

The ChIP-seq data of Pol II Ser2P, Pol II Ser5P, BRD4, H3K27ac, H3K27me3, and H3K4me1; CUT & Tag data of AR; RNA-seq data of 22Rv1 WT and *HOTAIR* poly-A knock-in cell lines (HKI-1, HKI-2); and ChIRP-seq data of *HOTAIR* have all been uploaded to GEO database (GSE 221263). Publicly available ChIP-seq data (AR, H3K27me3, H3K27ac) from prostate cancer tissues were downloaded from GEO (GSE130408).
