# Supplementary material for: HOTAIR Promotes Prostate Cancer Progression by facilitating CDK9-Pol II Association and Transcriptional Elongation
Source: Int J Biol Sci. 2026 Sep 10;22(14):8054–73. doi: 10.7150/ijbs.134766 (PMC13618222; doi:10.7150/ijbs.134766)
Supplement: Supplementary file 1 [file ijbsv22p8054s1.pdf]

Supplementary Materials for

HOTAIR Promotes Prostate Cancer Progression by facilitating CDK9–Pol II Association and Transcriptional Elongation

Yuyang Qian[1,4,+], Bo Wang[1,+], Guangsong Su[3], Peng Li[1], Zhenhao Zhao[1], Yuan Jiang[1], Lei Zhang[1], Zhongfang Zhao[1], Mulin Jun Li[2], Jiandang Shi[1,*], Dandan Huang[2,*], Wange Lu[1,3,*]

[1] State Key Laboratory of Medicinal Chemical Biology, Frontiers Science Center for Cell Responses, College of Life Sciences, Nankai University, Tianjin, PR China

[2] Department of Pharmacology, State Key Laboratory of Experimental Hematology, The Province and Ministry Co-sponsored Collaborative Innovation Center for Medical Epigenetics, Tianjin Key Laboratory of Inflammation Biology, School of Basic Medical Sciences, Tianjin Medical University, Tianjin, PR China

[3] Department of Laboratory Medicine and Institute of Precise Medicine, The First Affiliated Hospital, Sun Yat-sen University, Guangzhou, PR China

[4] Department of Head and Neck Oncology, Tianjin Medical University Cancer Institute &Hospital, Key Laboratory of Basic and Translational Medicine on Head & Neck Cancer, Tianjin, National Clinical Research Center for Cancer, Tianjin's Clinical Research Center for Cancer, Tianjin, PR China

[+]Yuyang Qian and Bo Wang contributed equally to this work.

***Correspondence**: Wange Lu: lvwg@mail.sysu.edu.cn; Dandan Huang: ddhuang@tmu.edu.cn; Jiandang Shi: shijd@nankai.edu.cn.

This file includes:

Additional file 1: Supplementary Figures: Figures S1 to S15.

Additional file 2: Supplementary Tables: Table S1 to S2.

Additional file 1:

Fig. S1. The 5’ RACE PCR and Sanger sequencing results of *HOTAIR* in PCa cell line.

Fig. S2. *HOTAIR* plays a critical role in the progression of PCa.

Fig. S3. *HOTAIR* over-expression rescues tumorigenesis in *HOTAIR* silenced PCa cells.

Fig. S4. Odd/even probe reproducibility assessment of HOTAIR ChIRP-seq peaks.

Fig. S5. Replicate-level signal correlations across assays and replicate-level QC summary.

Fig. S6. H3K27me3 and H3K27ac enrichment significantly changes while H3K4me1 enrichment no notable changes following *HOTAIR* silencing.

Fig. S7. Genomic distribution and gene expression changes near *HOTAIR*- targets in H3K27me3- regions after *HOTAIR* silencing.

Fig. S8. Cistrome support for HOTAIR-associated AR/Pol II/BRD4 co-localization.

Fig. S9. *HOTAIR* silencing drops only AR signal near its peak center.

Fig. S10. AR signal enrichment decreases across genomic loci after *HOTAIR* silencing.

Fig. S11. Full-length *HOTAIR*, but not the Δ1-442 mutant, rescued colony formation and invasive migration in *HOTAIR*-silenced PCa cells.

Fig. S12. Knockdown *AR* in AR-positive 22Rv1 and C4-2B cells.

Fig. S13. *HOTAIR* depletion is associated with adhesion- and ECM-related

transcriptional changes.

Fig. S14. *MMP14* and *TNFAIP2* over-expression rescues tumorigenesis in *HOTAIR* silenced PCa cells.

Fig. S15. *HOTAIR*-associated regions overlap with active and repressive chromatin features in public prostate tumor ChIP-seq datasets.

Additional file 2:

Table. S1. The colocalization of *HOTAIR* and prostate associated TFs.

Table. S2. Primers and oligos used in this study.

Figure S1

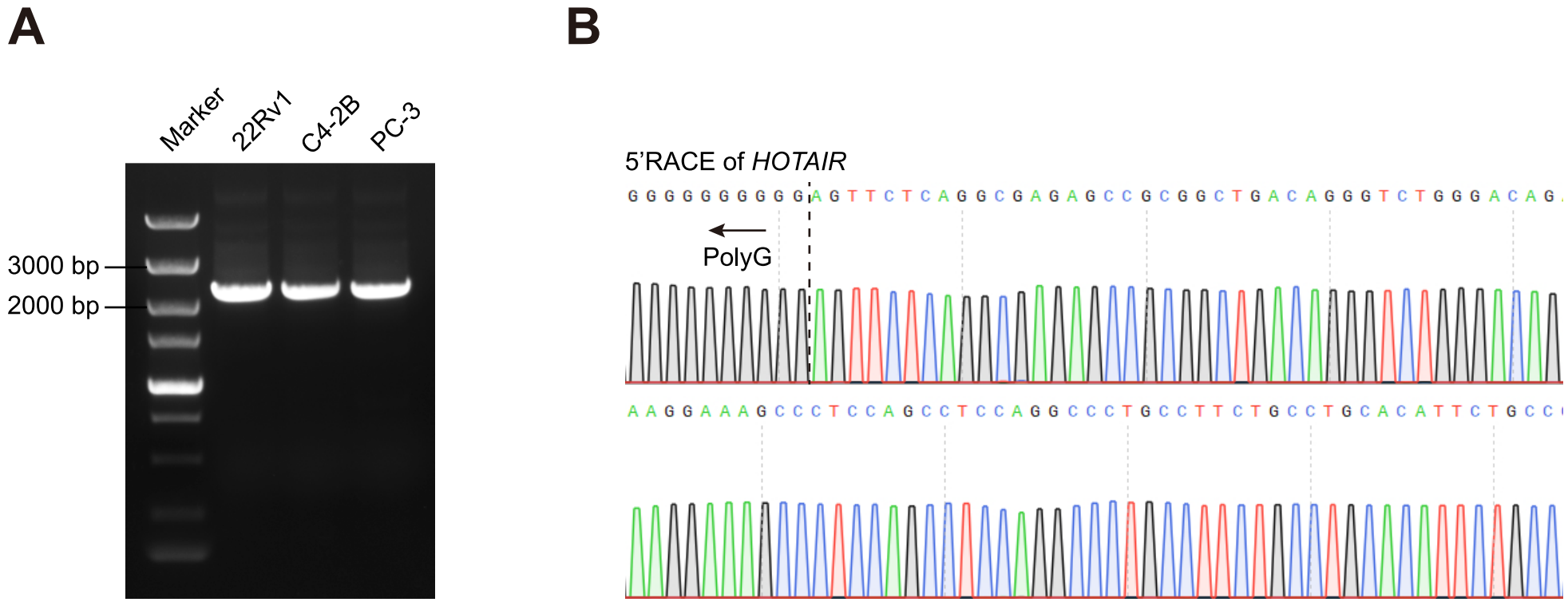

Fig S1. The 5′ RACE PCR and Sanger sequencing results of *HOTAIR* in PCa cell line.
(A) The gel shows the 5′ RACE amplifications of *HOTAIR* in 22Rv1, C4-2B and PC-3.
(B) The Sanger sequencing result of 5′ RACE, only part of the sequencing result is displayed.

Figure S2

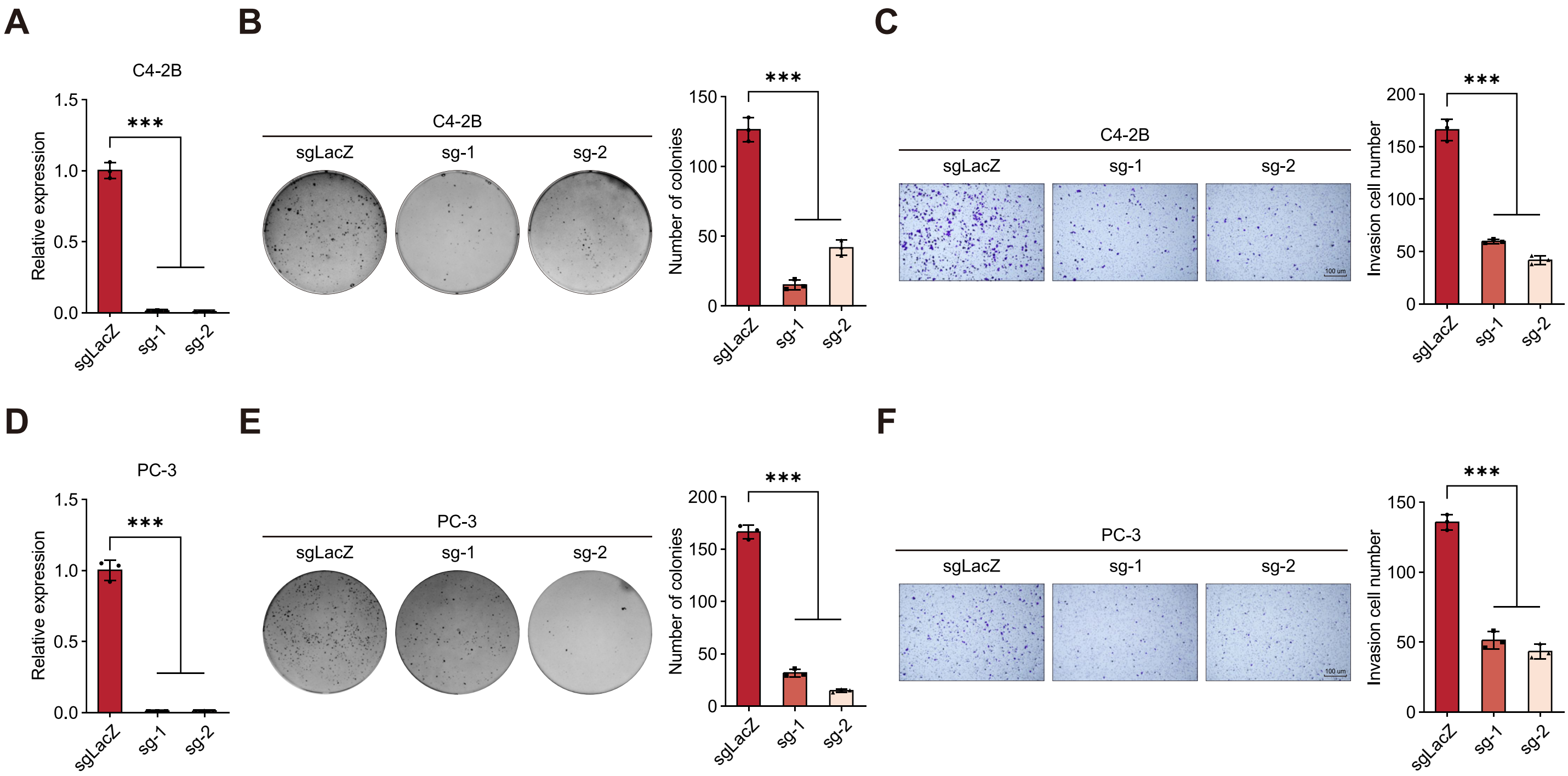

Fig S2. *HOTAIR* plays a critical role in the progression of PCa.

(A) RT-qPCR showing the expression of *HOTAIR* in the C4-2B WT cells (sgLacZ) and two *HOTAIR* CRISPRi cell lines (sg-1, sg-2). Data are shown as means ± SEM of three independent experiments (****P*<0.001).

(B) Soft agar assay comparing colony formation in the C4-2B WT cells (sgLacZ) and *HOTAIR* CRISPRi cell lines (sg-1, sg-2). Quantification of colony number are shown as means ± SEM of three independent experiments (****P*<0.001).

(C) Transwell assays indicating invasive migration by the C4-2B WT cells (sgLacZ) and *HOTAIR* CRISPRi cell lines (sg-1, sg-2). Quantification of migrated cells are shown as means ± SEM of three independent experiments (****P*<0.001).

(D) RT-qPCR showing the expression of *HOTAIR* in the PC-3 WT cells (sgLacZ) and two *HOTAIR* CRISPRi cell lines (sg-1, sg-2). Data are shown as means ± SEM of three independent experiments (****P*<0.001).

(E) Soft agar assay comparing colony formation in the PC-3 WT cells (sgLacZ) and *HOTAIR* CRISPRi cell lines (sg-1, sg-2). Quantification of colony number are shown as means ± SEM of three independent experiments (****P*<0.001).

(F) Transwell assays indicating invasive migration by the PC-3 WT cells (sgLacZ) and *HOTAIR* CRISPRi cell lines (sg-1, sg-2). Quantification of migrated cells are shown as means ± SEM of three independent experiments (****P*<0.001). Statistical significance was assessed using two-tailed Student's *t*-test.

Figure S3

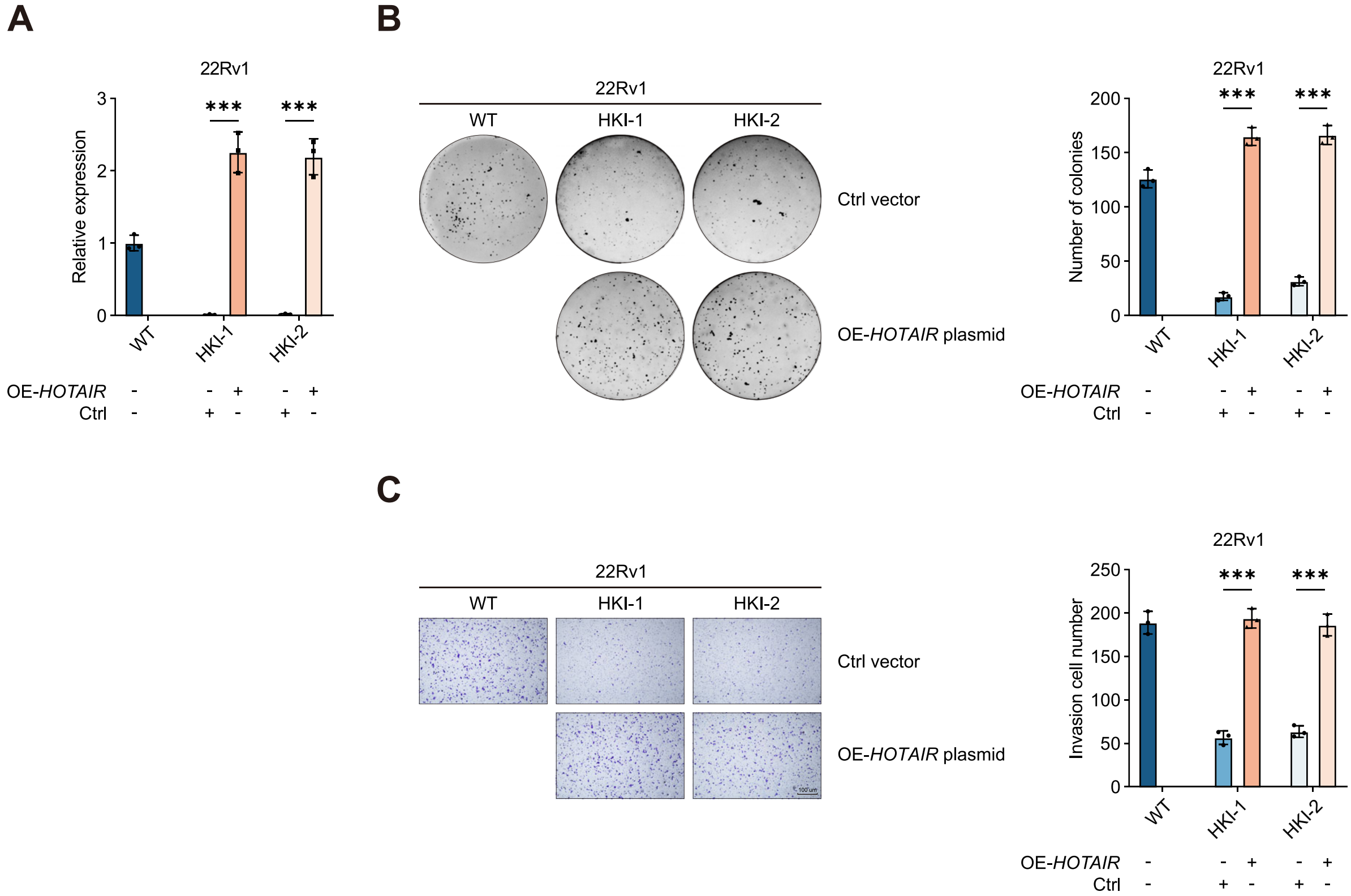

Fig S3. *HOTAIR* over-expression rescues tumorigenesis in *HOTAIR* silenced PCa cells.

(A) RT-qPCR analysis of *HOTAIR* in the 22Rv1 WT cells (WT), two poly-A knock-in lines (HKI-1, HKI-2) and poly-A knock-in lines re-expressing *HOTAIR* (OE-*HOTAIR*). Data are shown as means ± SEM of three independent experiments (****P*<0.001).

(B) Soft agar colony formation experiments for WT cells (WT), two poly-A knock-in lines (HKI-1, HKI-2) and poly-A knock-in lines re-expressing *HOTAIR* (OE-*HOTAIR*) in 22Rv1. Quantification of colony number are shown as means ± SEM of three independent experiments (****P*<0.001).

(C) Transwell assays for WT cells (WT), two poly-A knock-in lines (HKI-1, HKI-2) and poly-A knock-in lines re-expressing *HOTAIR* (OE-*HOTAIR*) in 22Rv1. Quantification of migrated cells are shown as means ± SEM of three independent experiments (****P*<0.001). Statistical significance was assessed using two-tailed Student's *t*-test.

Figure S4

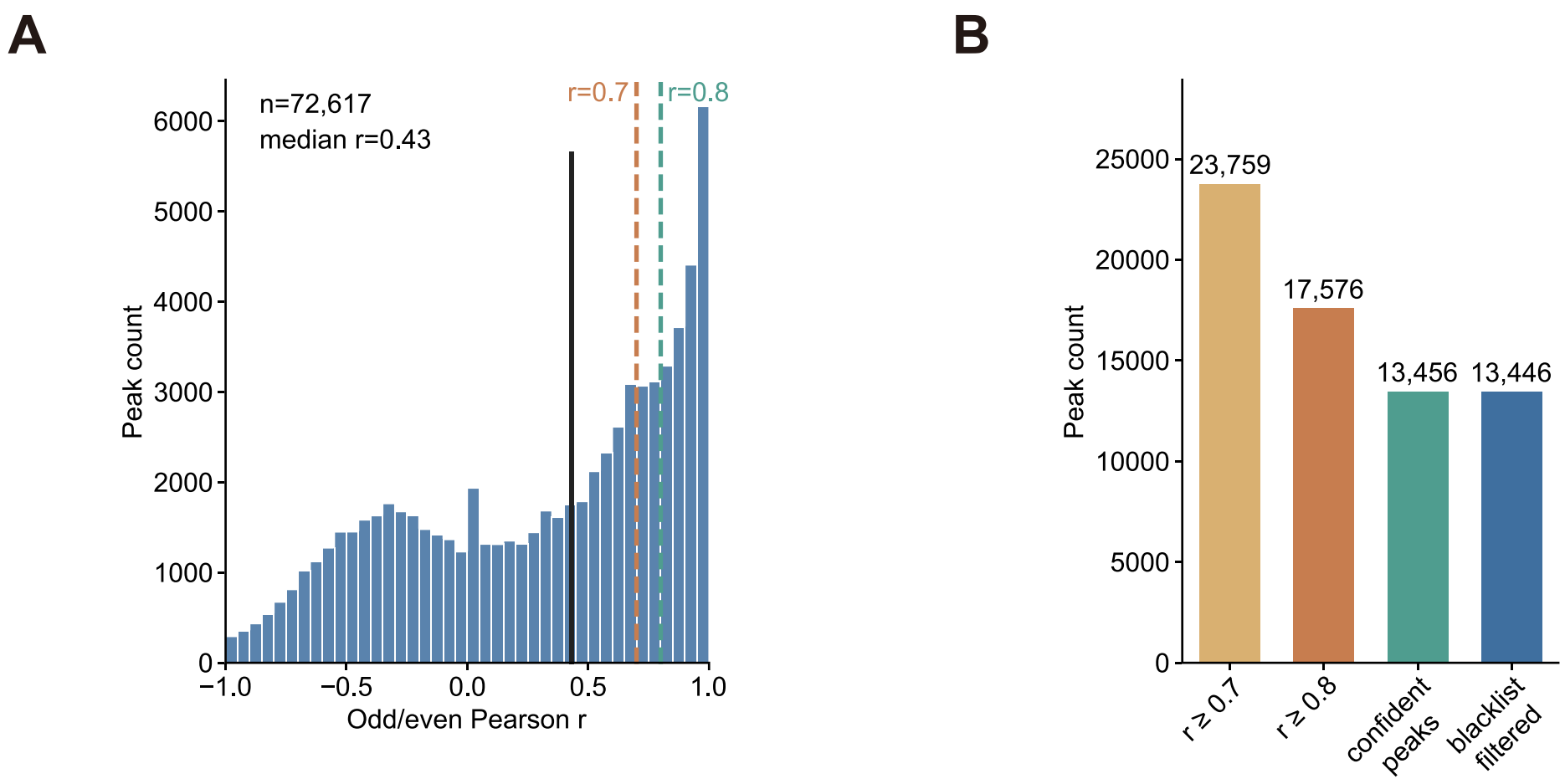

Fig S4. Odd/even probe reproducibility assessment of HOTAIR ChIRP-seq peaks.

(A) Distribution of Pearson correlation coefficients between odd and even HOTAIR ChIRP-seq probe pools across candidate HOTAIR-enriched peak regions. The total number of candidate peaks and the median odd/even correlation are indicated. Dashed lines mark representative reproducibility thresholds of r = 0.7 and r = 0.8.

(B) Number of retained HOTAIR peaks after sequential reproducibility and quality filtering, including peaks with odd/even Pearson r ≥ 0.7, peaks with r ≥ 0.8, confident peaks and confident peaks remaining after blacklist filtering. For sequencing data, statistical methods are described in Methods.

Figure S5

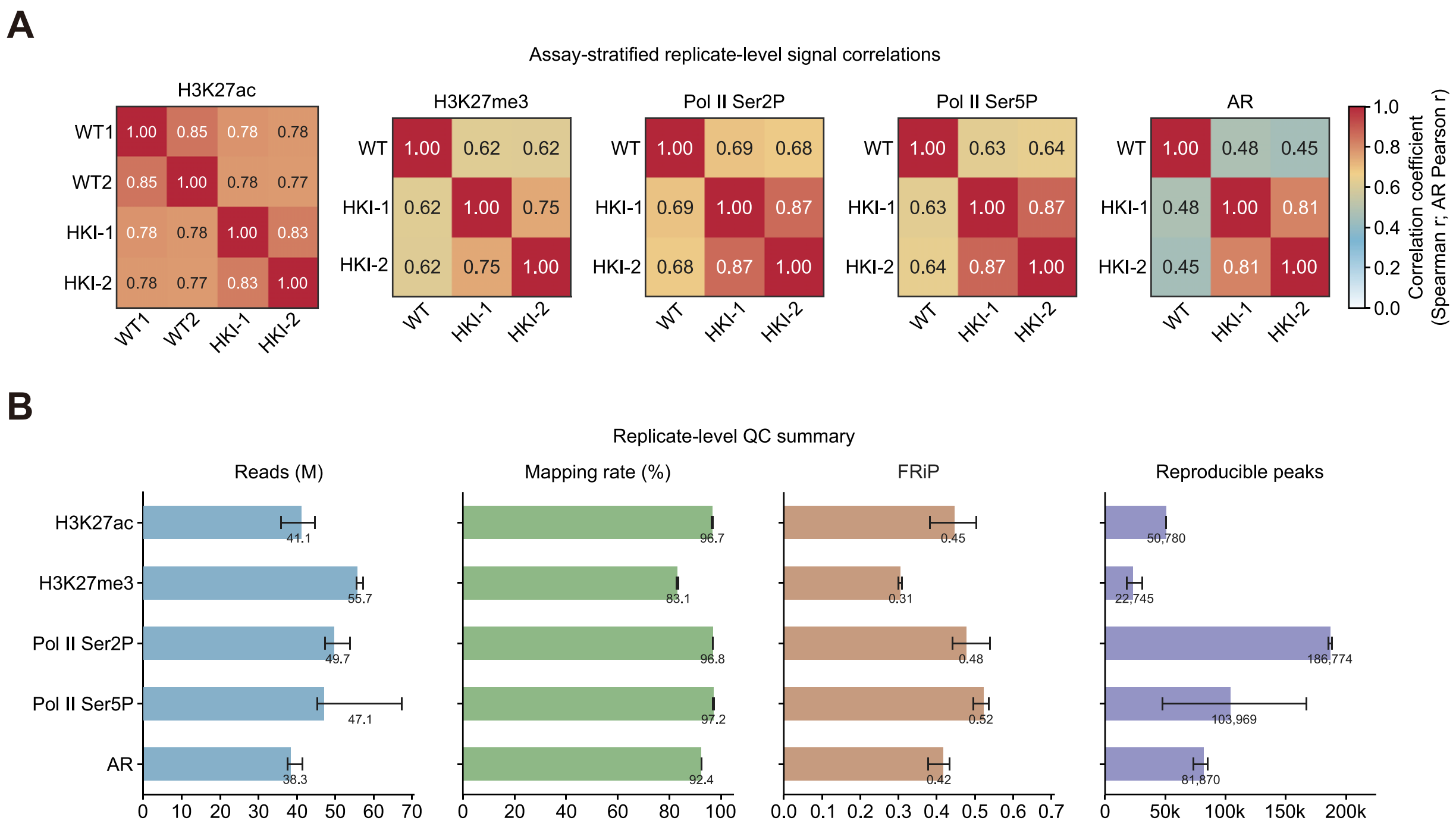

Fig S5. Replicate-level signal correlations across assays and replicate-level QC summary. (A) Replicate correlation heatmaps are shown for H3K27ac, H3K27me3, Pol II Ser2P, Pol II Ser5P, and AR. H3K27ac, H3K27me3, Pol II Ser2P, and Pol II Ser5P were summarized using Spearman correlation. Color indicates the pairwise correlation coefficient.

(B) Reads, mapping rate, FRiP, and reproducible peak counts are shown for the same assays used in the correlation overview. Bars indicate the median and error bars indicate the interquartile range. For sequencing data, statistical methods are described in Methods.

Figure S6

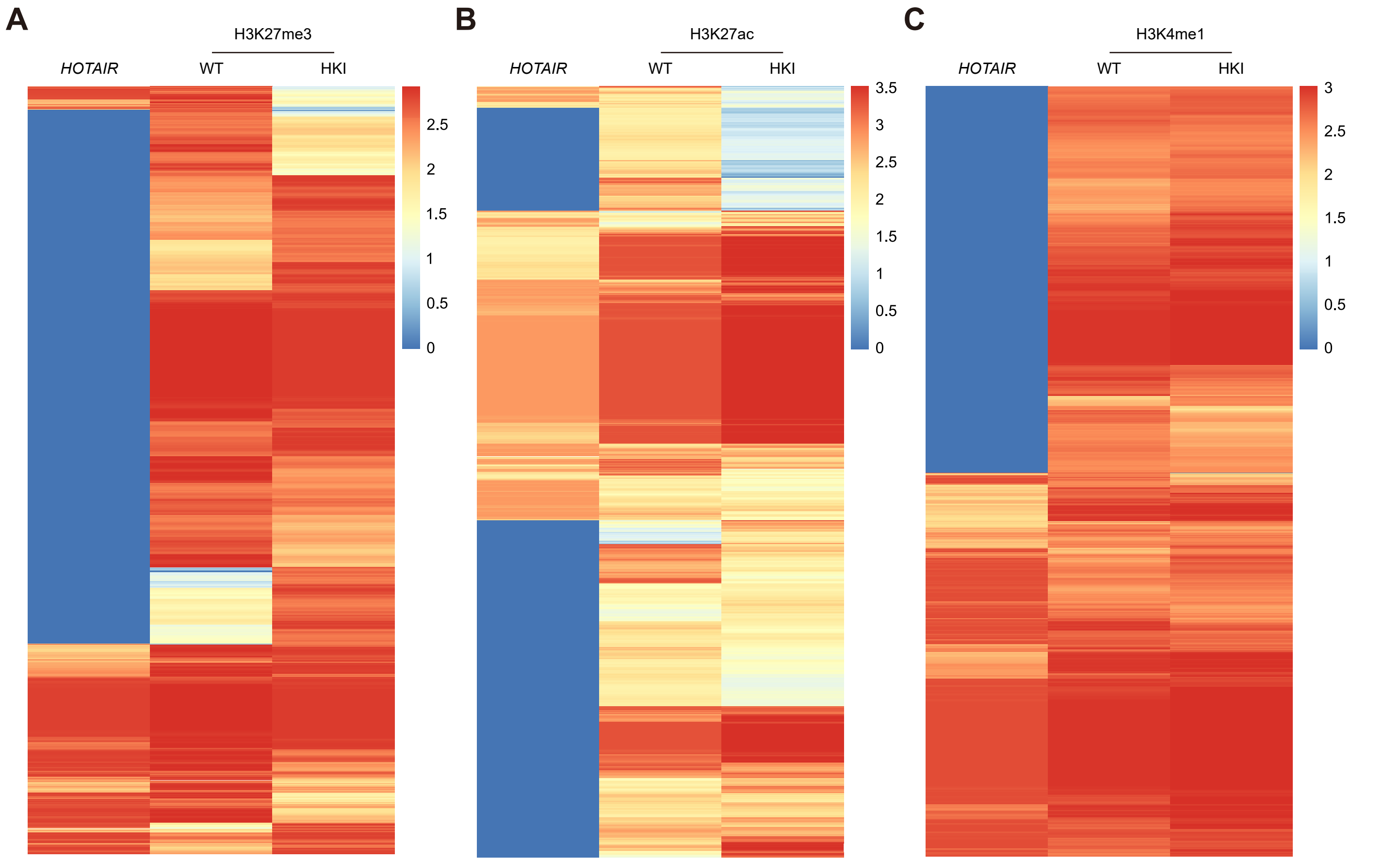

Fig S6. H3K27me3 and H3K27ac enrichment significantly changes while H3K4me1 enrichment no notable changes following *HOTAIR* silencing.

(A) Heatmap showing changes of enrichment of H3K27me3 signals around *HOTAIR* target and *HOTAIR* negative target regions after *HOTAIR* silencing (signal is defined as the average read density of each 2-kb bin). All HKI data represent pooled results from two biological replicates.

(B) Heatmap showing changes of enrichment of H3K27ac signals around *HOTAIR* target and *HOTAIR* negative target regions after *HOTAIR* silencing (signal is defined as the average read density of each 2-kb bin). All HKI data represent pooled results from two biological replicates.

(C) Heatmap showing changes in enrichment of H3K4me1 signals around *HOTAIR* target and *HOTAIR* negative target regions after *HOTAIR* silencing (signal is defined as the average read density of each 2-kb bin). All HKI data represent pooled results from two biological replicates. For sequencing data, statistical methods are described in Methods.

Figure S7

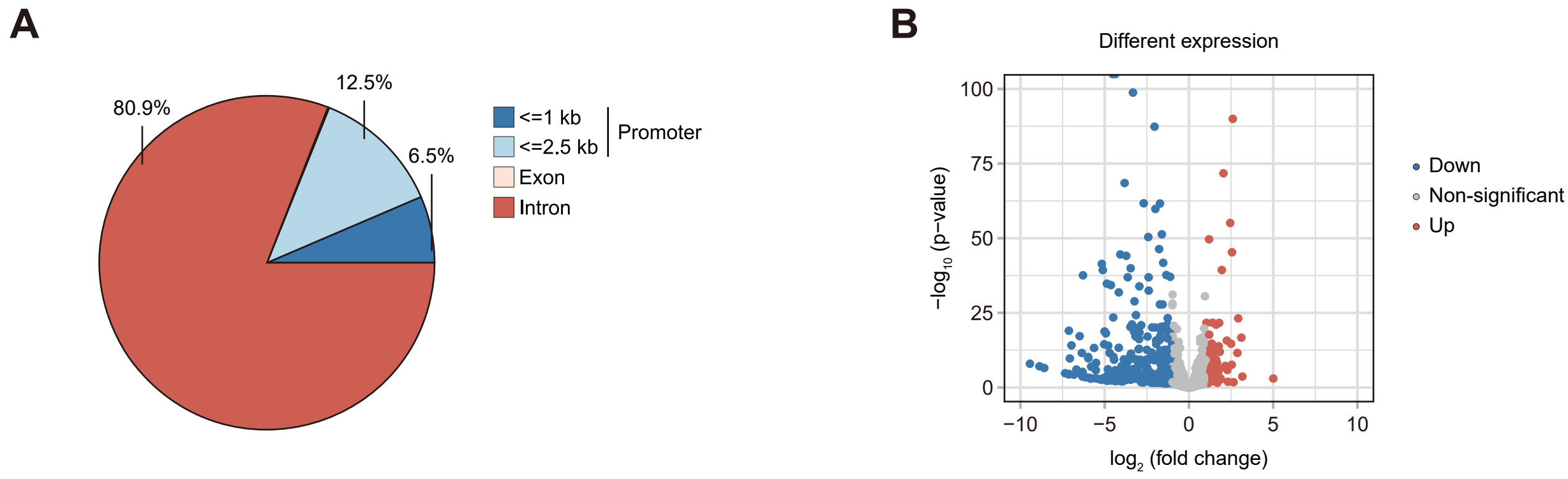

Fig S7. Genomic distribution and gene expression changes near *HOTAIR*- targets in H3K27me3- regions after *HOTAIR* silencing.

(A) Pie chart showing genomic distribution of the matched HOTAIR- group as defined in Figure 2A.

(B) Volcano plot depicting significantly changed genes in the matched HOTAIR- group as defined in Figure 2A. For sequencing data, statistical methods are described in Methods.

Firgue S8

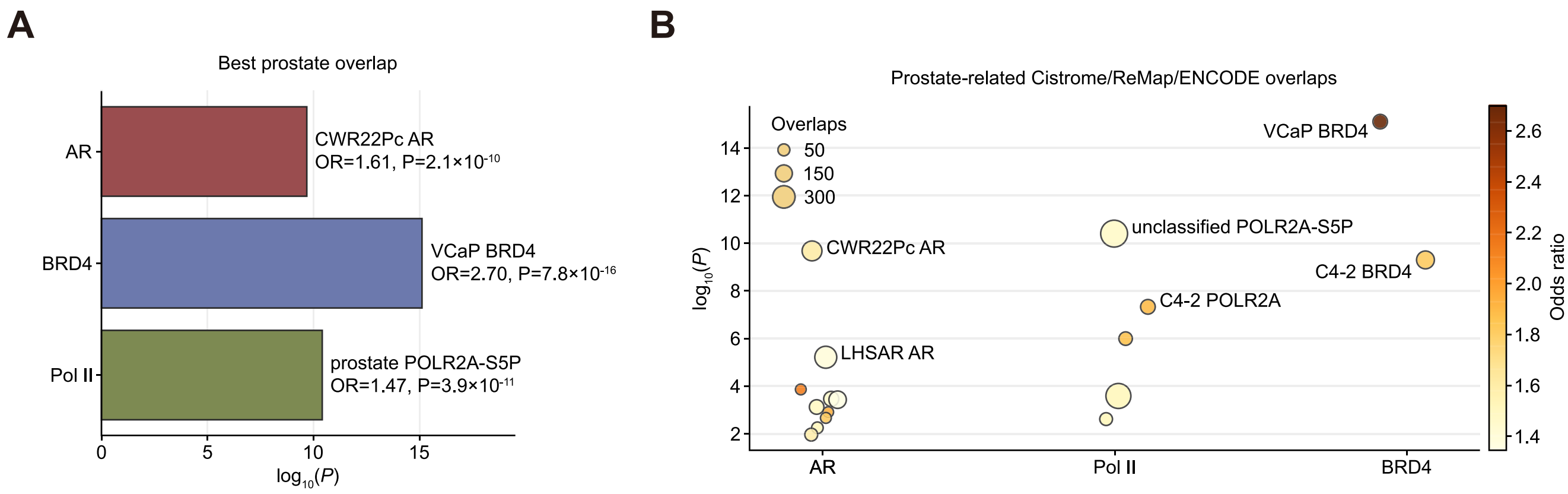

Fig S8. Cistrome support for HOTAIR-associated AR/Pol II/BRD4 co-localization.

(A) Best prostate-related Cistrome/ReMap/ENCODE overlap entries for AR, BRD4 and Pol II/POLR2A among HOTAIR ChIRP regions.

(B) Prostate-related overlap entries for the three factor classes. Dot position shows $-\log_{10}(P)$, dot size indicates overlap count and color indicates odds ratio. For sequencing data, statistical methods are described in Methods.

Figure S9

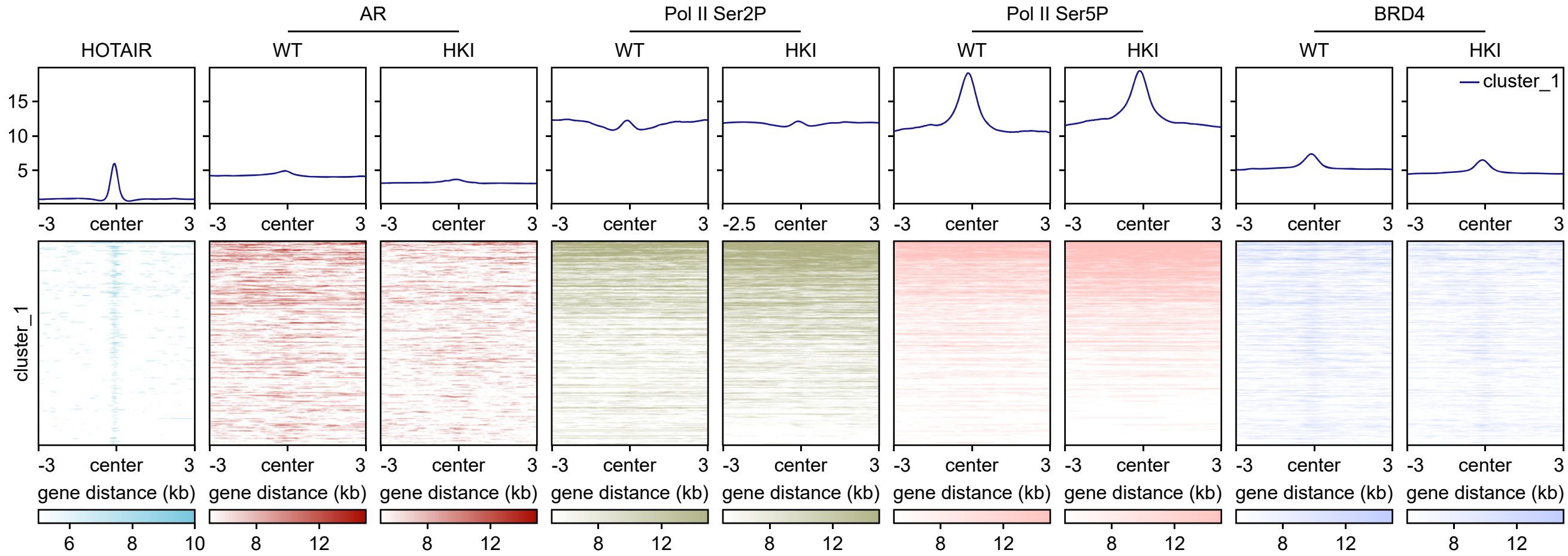

Fig S9. *HOTAIR* silencing drops only AR signal near its peak center.

Heatmaps generated from ChIRP-seq/ChIP-seq data showing enrichment of *HOTAIR*, AR, Pol II Ser2P, Pol II Ser5P, and BRD4 signals in WT and HKI lines. All rows are centered on *HOTAIR* ChIRP peaks. All HKI data represent pooled results from two biological replicates. For sequencing data, statistical methods are described in Methods.

Figure S10

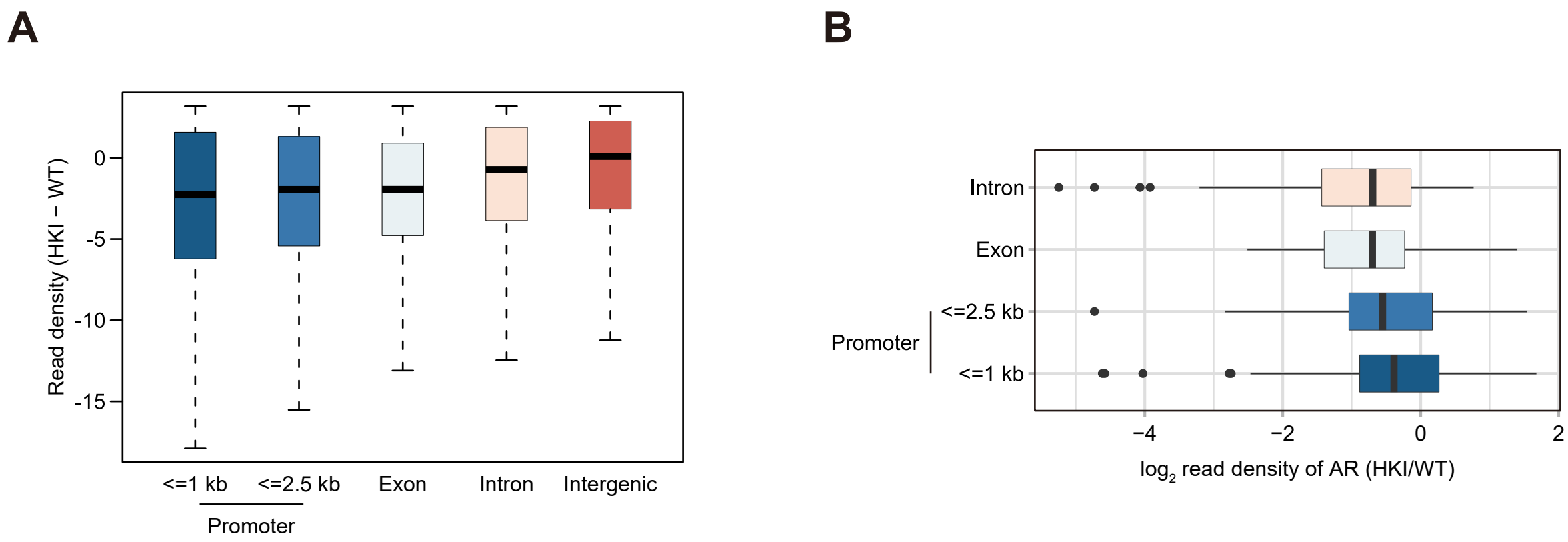

Fig S10. AR signal enrichment decreases across genomic loci after *HOTAIR* silencing.

(A) Boxplots showing absolute changes in AR read density after *HOTAIR* silencing across different genomic annotation categories. Values are calculated as HKI − WT read density. All HKI data represent pooled results from two biological replicates.

(B) Boxplots showing relative changes in AR read density around *HOTAIR* target regions after *HOTAIR* silencing across different genomic annotation categories. Values are shown as log_2-transformed HKI/WT read-density ratios. All HKI data represent pooled results from two biological replicates. For sequencing data, statistical methods are described in Methods.

Figure S11

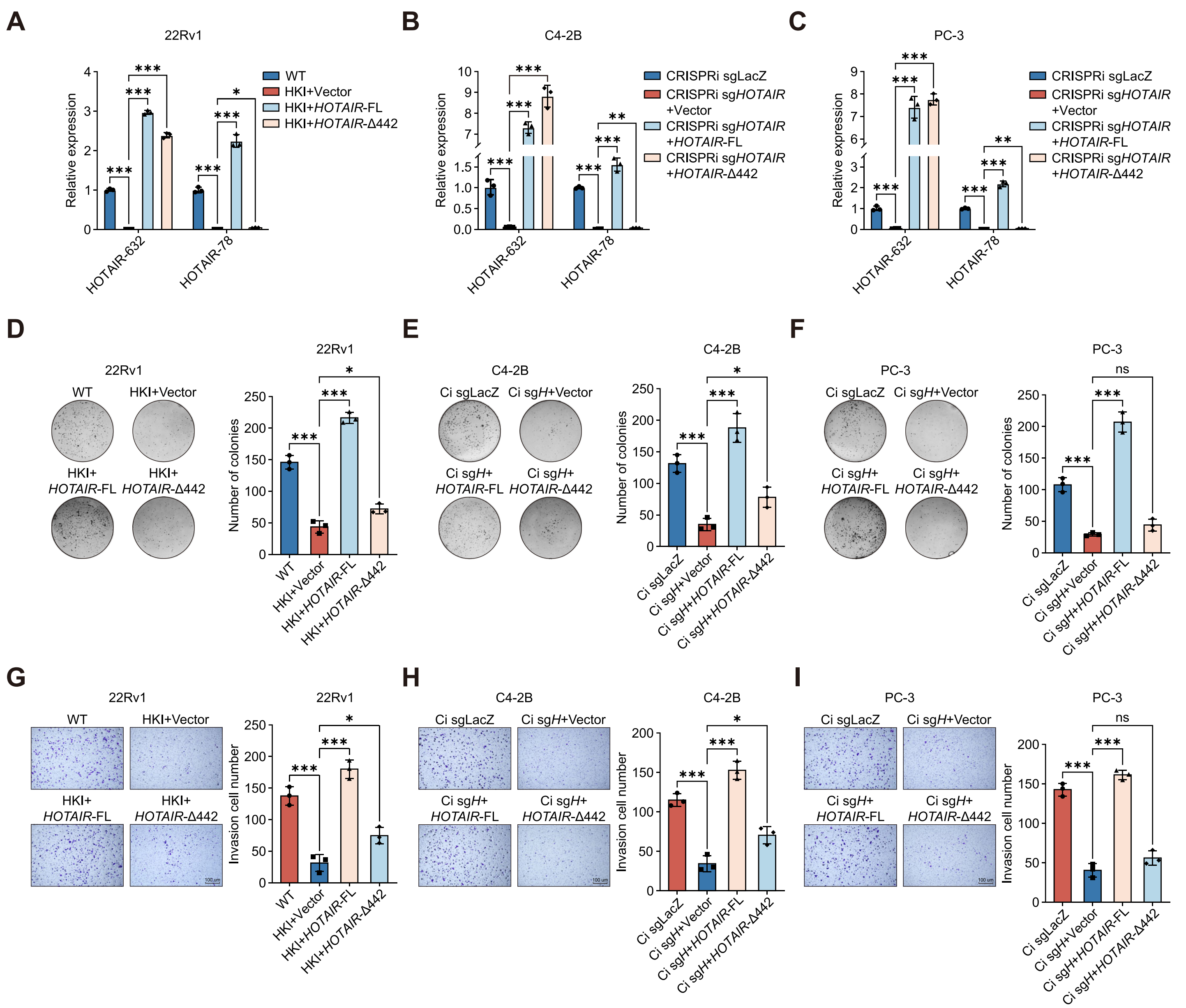

Fig S11. Full-length *HOTAIR*, but not the Δ1-442 mutant, rescued colony formation and invasive migration in *HOTAIR*-silenced PCa cells.

(A-C) RT-qPCR showing the expression of *HOTAIR* 443-2415 and *HOTAIR* 1-442 in WT 22Rv1, C4-2B and PC-3 cells and *HOTAIR*-silenced cells rescued with empty vector, full-length *HOTAIR*, or Δ1-442 mutant. The *HOTAIR*-632 primers detect both full-length and Δ1-442 transcripts, whereas the *HOTAIR*-78 primers detect only the full-length transcript. Data are shown as means ± SEM of three independent experiments (**P*<0.05, ***P*<0.01, ****P*<0.001).

(D-F) Soft agar assay comparing colony formation in WT 22Rv1, C4-2B and PC-3 cells and *HOTAIR*-silenced cells rescued with empty vector, full-length *HOTAIR*, or Δ1-442 mutant. Quantification of colony number are shown as means ± SEM of three independent experiments (**P*<0.05, ****P*<0.001; ns, not significant).

(G-I) Transwell assays indicating invasive migration by WT 22Rv1, C4-2B and PC-3 cells and *HOTAIR*-silenced cells rescued with empty vector, full-length *HOTAIR*, or Δ1-442 mutant. Quantification of migrated cells are shown as means ± SEM of three independent experiments (**P*<0.05, ****P*<0.001; ns, not significant). Statistical significance was assessed using two-tailed Student's *t*-test.

Figure S12

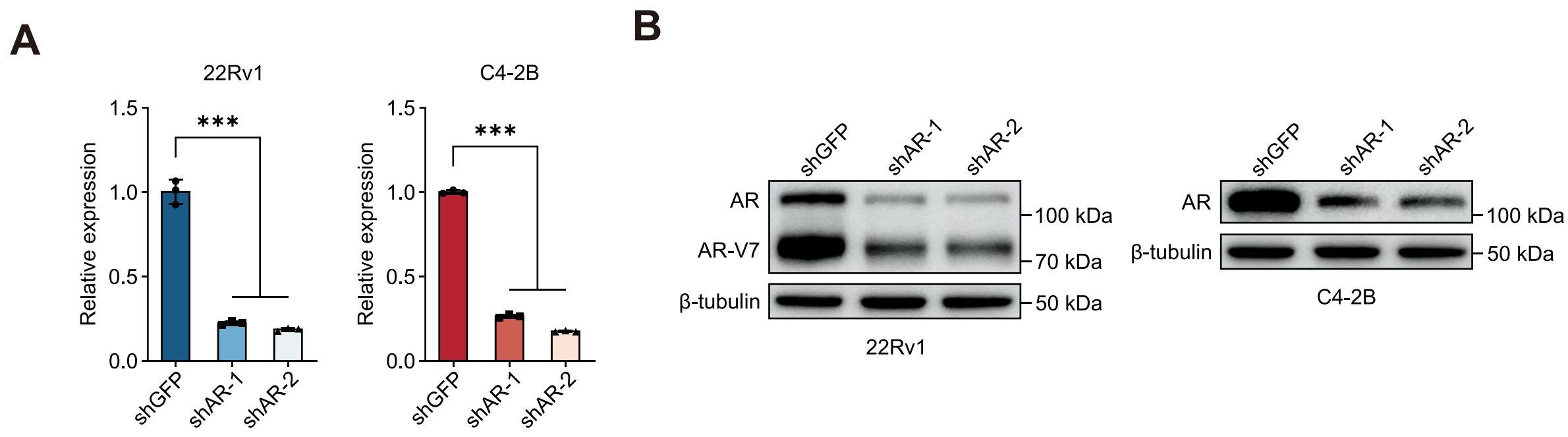

Fig S12. Knockdown *AR* in AR-positive 22Rv1 and C4-2B cells.

(A) RT-qPCR showing the expression of *AR* in AR-positive 22Rv1 and C4-2B cells following *AR* knockdown or shGFP. Data are shown as means ± SEM of three independent experiments (****P*<0.001). Statistical significance was assessed using two-tailed Student's *t*-test.

(B) Western Blot analysis of AR in AR-positive 22Rv1 and C4-2B cells following *AR* knockdown or shGFP.

Firgue S13

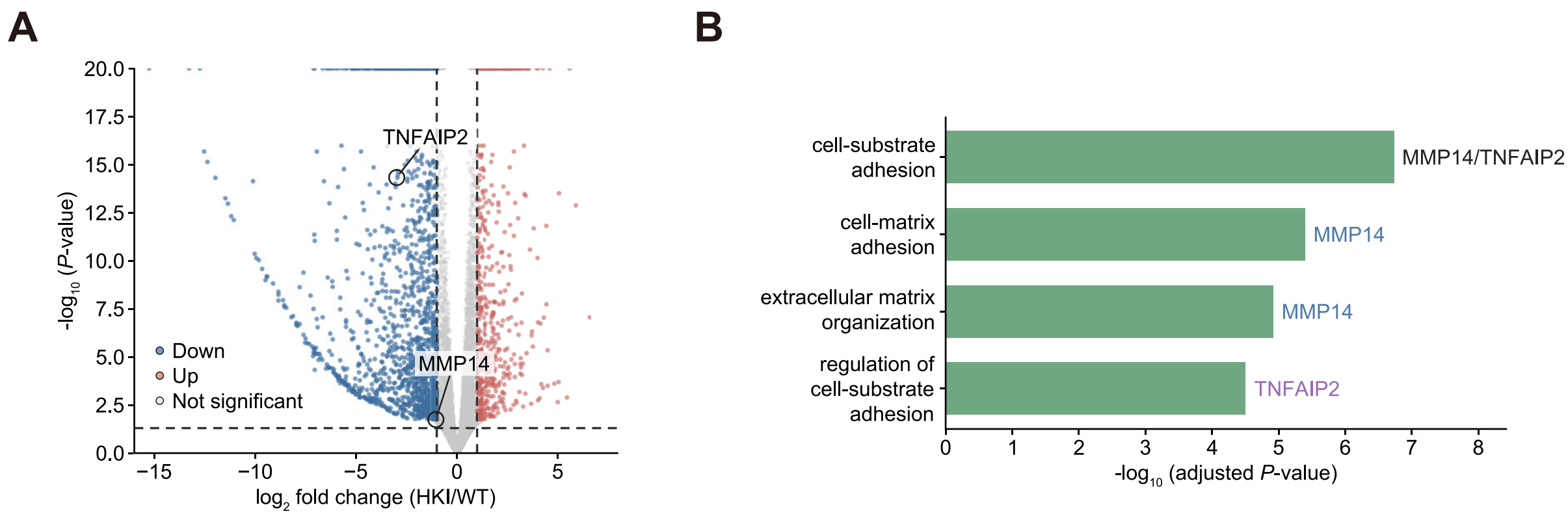

Fig S13. *HOTAIR* depletion is associated with adhesion- and ECM-related transcriptional changes.

(A) Volcano plot showing differential gene expression in *HOTAIR*-knockdown (HKI) cells compared with WT cells based on RNA-seq analysis. Red and blue dots indicate significantly upregulated and downregulated genes, respectively, and grey dots indicate non-significant genes. *TNFAIP2* and *MMP14* are highlighted.

(B) Gene ontology terms related to cell-substrate adhesion and extracellular matrix organization associated with the highlighted genes. Bar length indicates $-\log_{10}$-adjusted P value. These adhesion- and ECM-related annotations are consistent with the observed effects of *HOTAIR* depletion on cell adhesion and migration phenotypes. For sequencing data, statistical methods are described in Methods.

Figure S14

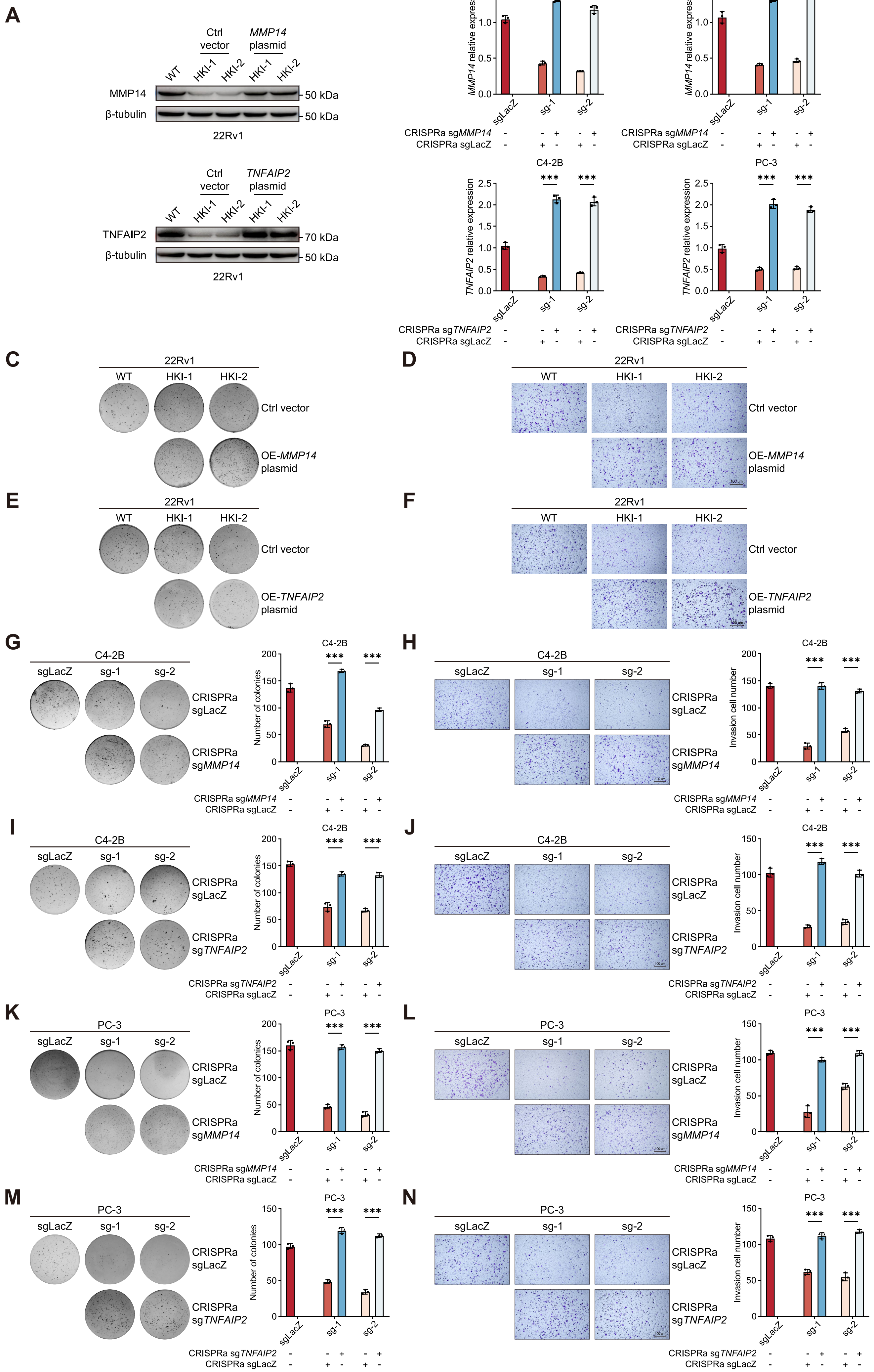

A
MMP14
β-tubulin
22Rv1
TNFAIP2
B
C4-2B
PC-3
MMP14 relative expression
TNFAIP2 relative expression
CRISPRa sgMMP14
CRISPRa sgTNFAIP2
CRISPRa sgLacZ
C
D
E
F
G
H
I
J
K
L
M
N
Ctrl vector
OE-MMP14 plasmid
OE-TNFAIP2 plasmid
Number of colonies
Invasion cell number

Figure S14

Fig S14. *MMP14* and *TNFAIP2* over-expression rescues tumorigenesis in *HOTAIR* silenced PCa cells.

(A) Western Blot analysis of MMP14 or TNFAIP2 in WT, two *HOTAIR* poly-A knock-in the 22Rv1 cell lines (HKI-1, HKI-2) and HKI cells re-expressing MMP14 or TNFAIP2.

(B) RT-qPCR analysis of *MMP14* or *TNFAIP2* in the C4-2B or PC-3 WT cells (sgLacz), two *HOTAIR* CRISPRi cell lines (sg-1，sg-2) and *HOTAIR* CRISPRi cells re-expressing (CRISPRa) *MMP14* or *TNFAIP2*. Data are shown as means ± SEM of three independent experiments (****P*<0.001).

(C, E) Soft agar colony formation experiments for the 22Rv1 WT cells, *HOTAIR* silenced cell lines and *HOTAIR* silenced cells re-expressing MMP14 or TNFAIP2 cells. The quantification data are shown in Figure 6 J, L.

(D, F) Transwell experiments for the 22Rv1 WT cells, *HOTAIR* silenced cell lines and *HOTAIR* silenced cells re-expressing MMP14 or TNFAIP2 cells. The quantification data are shown in Figure 6 K, M.

(G, I, K, M) Soft agar colony formation experiments for the WT cells, *HOTAIR* silenced cell lines and *HOTAIR* silenced cells re-expressing MMP14 or TNFAIP2 in two PCa cell lines (C4-2B and PC-3). Data are shown as means ± SEM of three independent experiments (****P*<0.001).

(H, J, L, N) Transwell experiments for the WT cells, *HOTAIR* silenced cell lines and *HOTAIR* silenced cells re-expressing MMP14 or TNFAIP2 in two PCa cell lines (C4-2B and PC-3). Data are shown as means ± SEM of three independent experiments (****P*<0.001). Statistical significance was assessed using two-tailed Student's *t*-test.

Figure S15

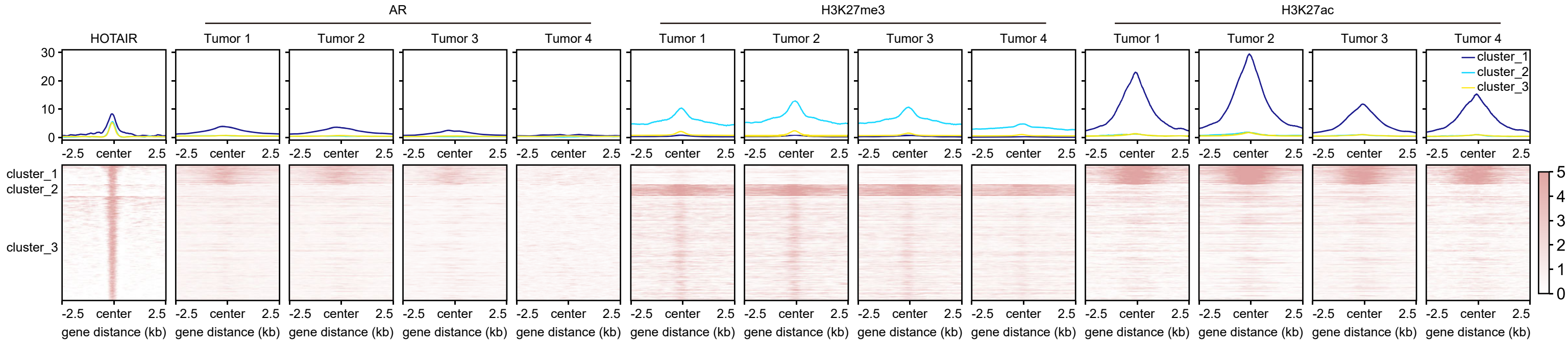

Fig S15. *HOTAIR*-associated regions overlap with active and repressive chromatin features in public prostate tumor ChIP-seq datasets.

Heatmaps showing HOTAIR ChIRP-seq signal from 22Rv1 WT cells together with public AR, H3K27ac, and H3K27me3 ChIP-seq profiles from four primary prostate tumor datasets. Rows are centered on HOTAIR ChIRP-seq peaks. For sequencing data, statistical methods are described in Methods.

Table. S1 The colocalization of *HOTAIR* and prostate associated TFs

GSM_ID	Factor	Biosource	GIGGLE_score
GSM2427948	SRC	Prostate	963.950832
GSM1868502	CHD4	LNCaP;Epithelium;Prostate	941.097644
GSM1868874	POLR2A	C4-2B;Epithelium;Prostate	932.68568
GSM2427951	AR	Prostate	783.286842
GSM1868875	POLR2A	C4-2B;Epithelium;Prostate	704.450393
GSM2427947	SRC	Prostate	594.543528
GSM2122804	AR	LNCaP;Epithelium;Prostate	572.973848
GSM2122805	AR	LNCaP;Epithelium;Prostate	558.358842
GSM2480826	TRIM28	LNCaP;Epithelium;Prostate	554.140809
GSM2480828	TRIM28	LNCaP;Epithelium;Prostate	491.852516
GSM2427945	AR	Prostate	439.105233
GSM1899558	ERG	VCaP;Epithelium;Prostate	420.174105
GSM2058884	POU2F1	LNCaP;Epithelium;Prostate	390.767363
GSM2427944	AR	Prostate	270.830226
GSM2219856	AR	LNCaP;Epithelium;Prostate	250.97623
GSM1328962	BRD4	VCaP;Epithelium;Prostate	220.675897
GSM2219885	RELA	LNCaP;Epithelium;Prostate	182.343896
GSM1586672	POLR2A	C4-2;Prostate	143.862606
GSM2277159	AR	22RV1;Epithelium;Prostate	139.753514
GSM984390	AR	22RV1;Epithelium;Prostate	138.134685
GSM2771597	BRD4	LREX';Epithelium;Prostate	137.532695
GSM1586673	POLR2A	C4-2;Prostate	137.35344

Table S2. Primers and oligos used in this study

Primer sequences used for 5' RACE of *HOTAIR*

Primer	Sequence
RACE GSP Primer	CACAAGGAATGTGCAGTCCT

Guide RNAs, KI test primers and donor oligos used to assess CRISRP/Cas9-mediated *HOTAIR* poly-A Knock in

Primer	Sequence
SgRNA	GCTTCCAGGTTCCGGAAATC
KI test forward	CATTTGTTTGGGATCTGTTCCAG
KI test reverse	CCTGAGCAGAAGGCACTTT
Donor oligos	TTCTGCCTGCACATTCTGCCCTGATAATAAAAGATCTTTATTTTCATTAGA TCTGTGTGTTGGTTTTTTGTGTGTTCCGGAACCTGGAAGCCTAGGCAG

Biotin-labeled probes used to *HOTAIR* ChIRP assay

Probe	Sequence
1	TGGCATTTCTGGTCTTGTAA
2	TTCGTGGTTGCTTTTTCTAC
3	GTCTTGTTAACAAGCCTCAT
4	ATCAATTAATTAGCGCCTCC
5	AAATCCAGAACCCTCTGACA
6	CCAACAAAACACACTCCCAA
7	CTATGTTCCTCTCAAATTCC
8	TCAAGAGCTTCCAAAGGCTA
9	ACTGCATAATCACTCCTGTA
10	TTTAAGTGTTCTCCTATGTC
11	TACCTACCCAATGTATGGAA
12	CCTGAGTCTATTTAGCTACA

Primers used for constructing in vitro transcription templates of truncated *HOTAIR*

Template names	Primer	Sequence
HOTAIR 1-442	T7-Forward	TAATACGACTCACTATAGGGAGTTCTCAGGCGAGAGCCGC
	Reverse	AAAGACGCCCCTCCTTCCTC
HOTAIR 443-743	T7-Forward	TAATACGACTCACTATAGGGATTTTTTTAAGGCCCCAAAG
	Reverse	TGCCGCCTTGCTCCCTTGCC
*HOTAIR*744-1632	T7-Forward	TAATACGACTCACTATAGGGGTTCCCGGAACAAACGTGG
	Reverse	TCACTTTTAAAAATTTGTTT
HOTAIR 1633-2415	T7-Forward	TAATACGACTCACTATAGGGACCAGCCCTAGCCTTTGGA
	Reverse	TTCACCACATGTAAAACTTT
HOTAIR anti-sense	T7-Forward	TAATACGACTCACTATAGGGTTCACCACATGTAAAACTTT
	Reverse	AGTTCTCAGGCGAGAGCCGC

Primers used for ChIRP-qPCR

Gene name	Primer	Sequence
MMP14	Forward	CTTTTGGGATCTCCGCTGCT
	Reverse	GGAATGGGAAGGAGAGTCGG
TNFAIP2	Forward	GCAGAGTTTTGGGTCCGAGA
	Reverse	GCGCTCGTGAATGAAATCCC
GAPDH	Forward	GTCGGAGTCAACGGATTTG
	Reverse	TGGGTGGAATCATATTGGAA

Primers used for ChIP-qPCR

Gene name	Primer	Sequence
MMP14	Forward	CTTTTGGGATCTCCGCTGCT
	Reverse	GGAATGGGAAGGAGAGTCGG
TNFAIP2	Forward	GCAGAGTTTTGGGTCCGAGA
	Reverse	GCGCTCGTGAATGAAATCCC

shRNA target sequences used for *AR* knockdown

shRNA targets	Sequence
shGFP	TTCTCCGAACGTGTCACGTTT
shAR-1	CGCGACTACTACAACTTTCCA
shAR-2	GAAATGTTATGAAGCAGGGAT

SgRNAs used for *HOTAIR* CRISPRi

SgRNAs	Sequence
SgLacZ	CCCGAATCTCTATCGTGCGG
Sg-1	CTCAACTTCTGACTGTAGTG
Sg-2	ACAGTGAGGACTGCTCCGTG

SgRNAs used for *MMP14* and *TNFAIP2* CRISPRa

SgRNAs	Sequence
sg*MMP14*	AATTACAACCAAGAACTGGA
sg*TNFAIP2*	CCGCAACCGCGGAAAACCCG

Primers used for RT-qPCR

Gene name	Primer	Sequence
HOTAIR	Forward	GGTAGAAAAAGCAACCACGAAGC
	Reverse	ACATAAACCTCTGTCTGTGAGTGCC
MMP14	Forward	CGAGGTGCCCTATGCCTAC
	Reverse	CTCGGCAGAGTCAAAGTGG
TNFAIP2	Forward	GGCCAATGTGAGGGAGTTGAT
	Reverse	CCCGCTTTATCTGTGAGCCC
HPRT1	Forward	CCTGGCGTCGTGATTAGTGAT
	Reverse	AGACGTTCAGTCCTGTCCATAA
AR	Forward	GACGACCAGATGGCTGTCATT
	Reverse	GGGCGAAGTAGAGCATCCT
HOTAIR-78	Forward	CCTGCACATTCTGCCCTGAT
	Reverse	CGGAGCAGGACCTTTCTGAT

Primers used for nascent RNA RT-qPCR

Gene name	Primer	Sequence
18s rRNA	Forward	GGAGTATGGTTGCAAAGCTGA
	Reverse	ATCTGTCAATCCTGTCCGTGT
MMP14	Forward	GGTGGTCTCGGACCATGTCT
	Reverse	CAAAAGCCCTCCTCTCCGAA
TNFAIP2	Forward	GCTGCCCCATTTTCAGAAGAC
	Reverse	CTGGGGATTGGGAAACGAGG
HPRT1	Forward	CATTTACCACTTCTAGGCCCC
	Reverse	TCAGTCCATAACAAGCACCC
